# Nanotechnological strategies to increase the oxygen content of the tumor

**DOI:** 10.3389/fphar.2023.1140362

**Published:** 2023-03-09

**Authors:** Junjie Zhang, Kaiyuan Tang, Runqi Fang, Jiaming Liu, Ming Liu, Jiayi Ma, Hui Wang, Meng Ding, Xiaoxiao Wang, Yanni Song, Dongliang Yang

**Affiliations:** ^1^ School of Fundamental Sciences, Bengbu Medical College, Bengbu, China; ^2^ Nanjing Stomatological Hospital, Medical School of Nanjing University, Nanjing, China; ^3^ Biochemical Engineering Research Center, School of Chemistry and Chemical Engineering, Anhui University of Technology, Ma’anshan, China; ^4^ Key Laboratory of Flexible Electronics (KLOFE), Institute of Advanced Materials (IAM), School of Physical and Mathematical Sciences, Nanjing Tech University (NanjingTech), Nanjing, China

**Keywords:** tumor microenvironment, hypoxia, nanomaterial, nanozyme, cancer treatment

## Abstract

Hypoxia is a negative prognostic indicator of solid tumors, which not only changes the survival state of tumors and increases their invasiveness but also remarkably reduces the sensitivity of tumors to treatments such as radiotherapy, chemotherapy and photodynamic therapy. Thus, developing therapeutic strategies to alleviate tumor hypoxia has recently been considered an extremely valuable target in oncology. In this review, nanotechnological strategies to elevate oxygen levels in tumor therapy in recent years are summarized, including (I) improving the hypoxic tumor microenvironment, (II) oxygen delivery to hypoxic tumors, and (III) oxygen generation in hypoxic tumors. Finally, the challenges and prospects of these nanotechnological strategies for alleviating tumor hypoxia are presented.

## 1 Introduction

Studies have shown that the distribution of oxygen concentration in tumors is highly heterogeneous, and the partial pressure of oxygen in many areas is less than 5 mmHg (equivalent to approximately 0.7% O_2_), which is far lower than the partial pressure of oxygen in normal tissues (Vaupel et al., 1991; Denny, 2000). There are three main causes of hypoxia in the tumor microenvironment (TME). First, the rate of tumor apoptosis is much lower than the rate of cell growth, and the demand for oxygen and glucose is much greater than that of normal cells (Petrova et al., 2018; Matuszewska et al., 2021). Second, the tumor volume is constantly increasing. When the tumor volume increases to the oxygen diffusion limit (100–200 μm), the blood vessels cannot provide sufficient oxygen for the tissue cells (Bennewith and Dedhar, 2011; Fu et al., 2022).

Hypoxia can alter the expression of some cytokines, such as erythropoietin and metabolism-related proteins (e.g., phosphofructokinase), in the TME. These changes play an important role in the adaptation of tumor cells to hypoxia, energy storage, metastasis, proliferation, and tumor angiogenesis and eventually lead to metabolic abnormalities in tumor cells, which also exacerbates the malignant growth of tumors and drug resistance (Zhao et al., 2015). Hypoxia-inducible factor 1 (HIF-1) is involved in the regulation of cytokines and metabolism-related proteins. HIF-1 is a nuclear protein with transcriptional activity that consists of HIF-1α subunits and HIF-1β subunits. HIF-1α was degraded by ubiquitination under atmospheric oxygen. HIF-1α remained stable under hypoxic conditions. HIF-1β is stably expressed in cells and plays a structural role. To exert the role of a transcription factor, the HIF-1α subunit must polymerize with the HIF-1β subunit to form a heterodimer. The gene that responds to hypoxia stress is called the hypoxia response gene (HRG), and the gene regulated by HIF-1α in HRG is the target gene of HIF-1α. The promoters or enhancers of these target genes contain different numbers of hypoxia response elements (HREs), which are the binding sites of HIF-1α. HIF-1α combines with HRE to form a transcription initiation complex, which initiates the transcription of target genes and produces various products, causing a series of reactions. For example, under hypoxic conditions, HIF-1 can bind to the HRE of the vascular endothelial growth factor (VEGF) promoter region, causing the upregulation of VEGF expression, increasing the generation of new blood vessels, and promoting the growth and metastasis of tumors (Zhang et al., 2022a; Zhang et al., 2022b).

**FIGURE 1:**
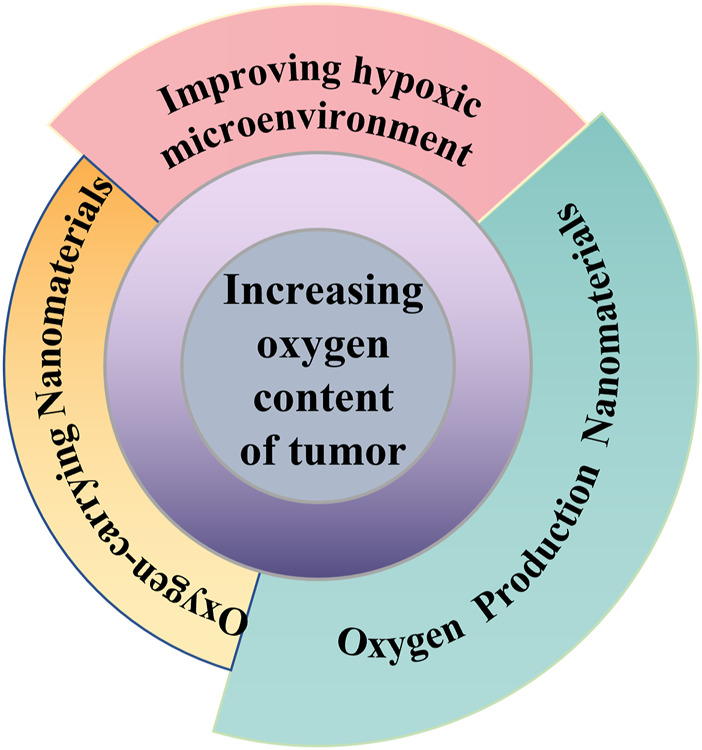
Schematic diagram of increasing oxygen content of tumor.

Hypoxia also promotes proteomic and genetic changes in tumor cells, reinforces the adaptation of cancer cells to hypoxia, and enhances tumor invasion and metastasis (Rankin and Giaccia, 2016a; Al Tameemi et al., 2019; Li et al., 2020a). Moreover, hypoxia has been proven to make tumor cells resistant to conventional cancer therapies, including chemotherapy, radiotherapy, and photodynamic therapy, which is due to the need for oxygen molecules to participate in these treatments (Yuan et al., 2021a; Devarajan et al., 2021; Kopecka et al., 2021; Telarovic et al., 2021). Therefore, relieving tumor hypoxia is the key to achieving effective cancer therapy. To improve the hypoxic microenvironment, many trials have been performed. For example, hyperbaric oxygen therapy increases oxygen in the blood and tumor by delivering high concentrations of oxygen to the body (Wu et al., 2018). However, non-specific oxygen delivery and severe malformations of the tumor microvasculature have prevented further development of hypoxia palliative therapies. In addition, high concentrations of oxygen may cause serious side effects (Schwarte et al., 2019). Fortunately, the rapid development of nanotechnology and materials science has led to tremendous progress in the biological applications of nanomaterials for molecular imaging, targeted drug delivery and combination therapy. There is growing evidence that nanomedicines offer many advantages in the treatment of hypoxic tumors. For example, by virtue of the tumor-specific enhanced permeability and retention properties, nanocarriers can be enriched in tumors (Gao et al., 2019; Yan et al., 2021; Yan et al., 2022). Then, nanomaterials with oxygen release ability can alleviate tumor hypoxia (Dai Phung et al., 2020; Zou et al., 2021; Xu et al., 2022a). In this review, we discuss the recent nanotechnologies to increase oxygen levels in tumors, including (I) improvement of the hypoxic TME, (II) oxygen-carrying nanomaterials, and (III) oxygen-production nanomaterials (Figure 1; Table 1). Finally, we present in detail the challenges and prospects of these tactics for alleviating tumor hypoxia.

**TABLE 1:** Nanotechnological strategies to increase the oxygen content of tumors.

Strategies	Nanomaterials to increase oxygen content	References
Improving the hypoxic TME	Nanomaterials combined antiangiogenic agents	Li et al. (2016)
	Inhibition of the HIF-1 signaling pathway based on nanomaterials	Kopecka et al. (2015), Zhu et al. (2015), Liu et al. (2016a), Liu et al. (2019)
	Photothermal therapy-mediated hypoxia relief	Lu et al. (2018), Li et al. (2020b), Li et al. (2022a), Zhang et al. (2022c)
Oxygen-carrying nanomaterials	Hemoglobin-based oxygen nanocarriers	Tian et al. (2017), Wang et al. (2021a), Yuan et al. (2021b)
	Perfluorocarbon-based oxygen nanocarriers	Song et al. (2016), Yang et al. (2021), Zhang et al. (2022d)
	Monocytes-based oxygen nanocarriers	Huang et al. (2015)
Oxygen-production nanomaterials	Catalase-loaded nanoagents	Qin et al. (2021), Zai et al. (2021), Wu et al. (2022), Zhou and Li (2022)
	Catalase-like nanozymes	He et al. (2013), Liu et al. (2017a), Yang et al. (2017), Jiang et al. (2019), Xu et al. (2019), Liu et al. (2020a), You et al. (2020a), Zhou et al. (2020a), Zhou et al. (2020b), Gao et al. (2020), Lu et al. (2020), Wei et al. (2020), Wei et al. (2021), Wang et al. (2022a), Xu et al. (2022b), Chang et al. (2022), Zhang et al. (2022e), Hou et al. (2022), Tian et al. (2022), Yu et al. (2022)
	Microalgae-based oxygen generators	Zheng et al. (2018), Qiao et al. (2020), Zhong et al. (2020), Wang et al. (2022b), Wang et al. (2022c)
	Metal peroxides-based oxygen generators	Chen et al. (2022a), Wang et al. (2022d), Koo et al. (2022)

## 2 Nanomaterial-mediated tumor hypoxia relief

### 2.1 Improving the hypoxic tumor microenvironment

Most solid tumors remain in a hypoxic state throughout disease progression (Chen et al., 2022b). For their own development, tumors will plunder nutrients and oxygen through angiogenesis (Chen et al., 2020). Tumor blood vessels differ from normal blood vessels in various important phenotypes. The expression of hypoxia-inducible angiogenic factors, such as VEGF, which induces angiogenesis *in vivo*, is upregulated in hypoxic tumor sites. However, the newly formed vessels tend to be poorly organized and dysfunctional, with either variable flow directions or leakage. In addition, there are gaps between tumor-related vessel endothelial cells, which cannot complete the normal metabolic exchange of plasma and tissue fluid, resulting in leakage of blood vessels and no laminar flow, which is prone to coagulation and local tissue edema. The hypoxic tumor environment is the main cause of tumor angiogenesis, and angiogenesis is the root cause of tumor progression and metastasis. Therefore, improving the hypoxic tumor environment is a conservative tumor treatment method that can inhibit tumor progression and metastasis (Wang et al., 2020a).

#### 2.1.1 Nanomaterials combined antiangiogenic agents

Some antiangiogenic agents normalize the vasculature by improving tumor blood flow and correspondingly delivering more oxygen to the tumor (Huang and Chen, 2010; Carmeliet and Jain, 2011). Normalizing the abnormal blood vessels in breast tumors by blocking vascular endothelial growth factor receptor 2 can facilitate the delivery of small nanoparticles (12 nm) while hindering the delivery of large nanoparticles (125 nm) (Chauhan et al., 2012). Antiangiogenic agents can increase tumor oxygen content by normalizing tumor blood supply, while normalization of vascular structure also enhances the delivery of nanodrugs, so antiangiogenic agents can synergize with nanodrugs in tumors. Li et al. (2016) used gold nanocarriers (Au NPs) to encapsulate recombinant human endostatin (rhES) to target tumors to enhance the antitumour effect. Polyethylene glycol (PEG)-modified rhES-Au-NPs (rhES-Au-NPs-PEG) can transiently normalize blood vessels and improve blood supply capacity in mouse-loaded hepatoma-22 xenografts. Then, tumor hypoxia relief, decreased vascular permeability, enhanced integrity, improved pericyte coverage, and increased blood perfusion were observed in the TME. The results of animal experiments proved that the combined treatment of rhES-Au-NPs-PEG and 5-fluorouracil was significantly more effective than the drug alone. In addition, endostatin and other antiangiogenic drugs, such as bevacizumab (anti-VEGF inhibitor), can also normalize tumor blood vessels (Isaakidou et al., 2016; Poluzzi et al., 2016).

#### 2.1.2 Modulation of relevant signal pathways for tumor hypoxia based on nanomaterials

Hypoxia causes a reduction in the enzyme activity of prolyl hydroxylase and prevents HIF-1α hydroxylation, which is the master transcriptional regulator in cells under hypoxic conditions (Gilkes et al., 2014; Rankin and Giaccia, 2016b; Jing et al., 2019; Singleton et al., 2021). The stability and activity of HIF-lα depends on oxygen content and affects tumor angiogenesis, glucose metabolism, tumor stem cell proliferation, tumor cell proliferation, and multidrug resistance (Fong and Takeda, 2008; Belisario et al., 2020). In the presence of 21% oxygen, the half-life of the HIF-1α subunit was less than 5 min, but when the O_2_ concentration was reduced to 1%, the half-life increased to 60 min. In the hypoxic TME, HIF-1α was no longer degraded and became stable. It binds to HIF-1β to form transcriptionally active dimers, which interact with transcriptional activation factors and finally induce the transcription and post-transcriptional regulation of downstream target genes, including erythropoiesis, glucose metabolism, angiogenesis, and drug resistance (Vaupel and Multhoff, 2018; McAleese et al., 2021). Therefore, inhibiting the HIF-1 signaling pathway is a suitable treatment strategy for hypoxic tumors. Researchers have developed several strategies to downregulate HIF-1α expression by using small interfering RNA (siRNA) (Cui et al., 2022; Huang et al., 2022; Zhou et al., 2022). However, there are some obstacles to siRNA therapeutics in systemic administration, such as aggregation with serum proteins, enzymatic degradation with endogenous nucleases, and uptake by phagocytes. In addition, the distance from the hypoxic area of the solid tumor to the blood vessel and the increase in efflux transporters also increase the difficulty of siRNA delivery. Zhu et al. (2015) designed a delivery system to deliver siHIF-1α. Hybrid quantum dots with hypoxic tumor targeting properties and pH responsiveness can enhance antitumour activity and reduce toxicity to normal tissues. In addition, this siRNA delivery system based on hybrid quantum dots can realize real-time dynamic monitoring of the siRNA delivery process *in vitro* and *in vivo*. Furthermore, the delivery of drugs that downregulate HIF-1α can also reverse tumor hypoxia tolerance (Liu et al., 2019). For example, zoledronic acid (ZA) can reduce the expression of P-glycoprotein (P-gp) in multidrug-resistant (MDR) cells and inhibit P-gp transcription mediated by HIF-1α (O'Donnell et al., 2006; Riganti et al., 2013). However, when ZA is administered directly, most ZA is absorbed by bone, resulting in insufficient concentrations in the tumor. To enhance the delivery effect, Kopecka et al. (2015) coupled ZA and liposomes to form nanoparticle formulations (abbreviated as NZ). The results have shown that NZ at low concentrations increases the chemical susceptibility of otherwise multidrug-resistant cells to commonly used broad-spectrum drugs. NZ can inhibit HIF-la activity by interfering with the Ras/ERK1 pathway. In addition, NZ can also reduce the transcription of glycolytic enzymes and glucose flux. Therefore, NZ reduced the activity of adenosine triphosphate-dependent adenosine triphosphate-binding transporters, thereby increasing the efficacy of chemotherapy *in vitro* and *in vivo*. To increase drug delivery efficiency, Liu et al. (2016a) utilized polyethylene glycol-poly L-lysine-poly lactic-co-glycollic acid to form nanoparticles through self-assembly, and then transferrin (Tf) was used for surface modification and loaded with daunorubicin (abbreviated as DNR-Tf-NPs) to study the antitumor effect on K562 leukemia cells under hypoxia. DNR-Tf-NPs could increase the intracellular concentration of daunorubicin (DNR) and the drug sensitivity of K562 cells. At the same time, DNR-Tf-NPs could reduce the expression of HIF-la, overcome multidrug resistance induced by hypoxia and induce apoptosis. In the xenograft model, DNR-Tf-NPs remarkably inhibited the growth of tumors and significantly reduced the expression of the Ki67 proliferation marker compared with other experimental groups.

#### 2.1.3 Photothermal therapy-mediated hypoxia relief

When the local temperature of the tumor tissue increases, the flow velocity increases. In recent years, a variety of nanomaterials with photothermal conversion ability have been used for tumor photothermal therapy, which could be enriched in the tumor site by passive targeting (based on the high permeability and retention effect of the tumor, i.e., the EPR effect) or active targeting (based on the binding of nanoparticle surface-coupled antibodies and tumor site-specific receptors) after being injected into mice. Under light irradiation, the local temperature of the tumor site increases locally, thereby achieving the purpose of treating and even killing tumors (Cheng et al., 2014). Based on the photothermal effect of MnS@Bi_2_Se_3_ nanomaterials, Song et al. (2015) found that a mild temperature rise (approximately 43°C) of the tumor site could be achieved under low power illumination. Interestingly, after mild photothermal treatment, the tumors were not killed, but the blood supply to the tumors could be significantly increased after a period of light exposure; thus, tumors would change from a hypoxic state to a relatively oxygen-rich state. Thus, the treated tumor could be effectively killed after oxygen-dependent X-ray radiotherapy, which was closely related to the significant increase in oxygen content inside the tumor before radiotherapy. In addition, Lu et al. (2018) synthesized CuS-modified hollow mesoporous organic silicon oxide nanoparticles (HMON@CuS) by loading oxygen-combined perfluoropentane (PFP). The heat generated by CuS after near infrared laser irradiation could convert the liquid PFP into a gaseous state and release it from the cavity of the HMON to further promote the release of oxygen and promote the diffusion of oxygen in the tumor, thereby overcoming the radiotherapy tolerance of the tumor. In addition, photothermal therapy combined with other treatment methods also proves that mild phototherapy can indeed lead to the improvement of blood supply and hypoxia in tumors, thus making tumors more sensitive to treatments (Li et al., 2020b; Li et al., 2022a; Zhang et al., 2022c).

### 2.2 Oxygen-carrying nanocarriers

The development of efficient oxygen delivery strategies is valuable and significant for enhancing the effect of tumor therapy. At present, hyperbaric oxygen is inhaled and used clinically during tumor treatment to force more oxygen into the blood and tumors (Chu et al., 2018; Liu et al., 2021; Li et al., 2022b). However, this strategy cannot overcome the defect of insufficient oxygen supply caused by vessel structural abnormity, and it is difficult to effectively transport oxygen to the interior of the tumor. Moreover, excessive oxygen concentration in the body is prone to oxygen toxicity to the lungs and central nervous system. In recent years, artificial oxygen carriers have become increasingly widely used. Artificial oxygen carriers usually use perfluorocarbons, hemoglobin or nanomaterials to store oxygen molecules inside the carrier by dissolving or non-covalent binding. When they reach the tumor, they can release the stored oxygen into the tumor tissue, thereby increasing the oxygen content of the tumor. Next, we discuss hemoglobin/perfluorocarbon-based nanocarriers to directly deliver oxygen to hypoxic tumors.

#### 2.2.1 Hemoglobin-based oxygen nanocarriers

**FIGURE 2:**
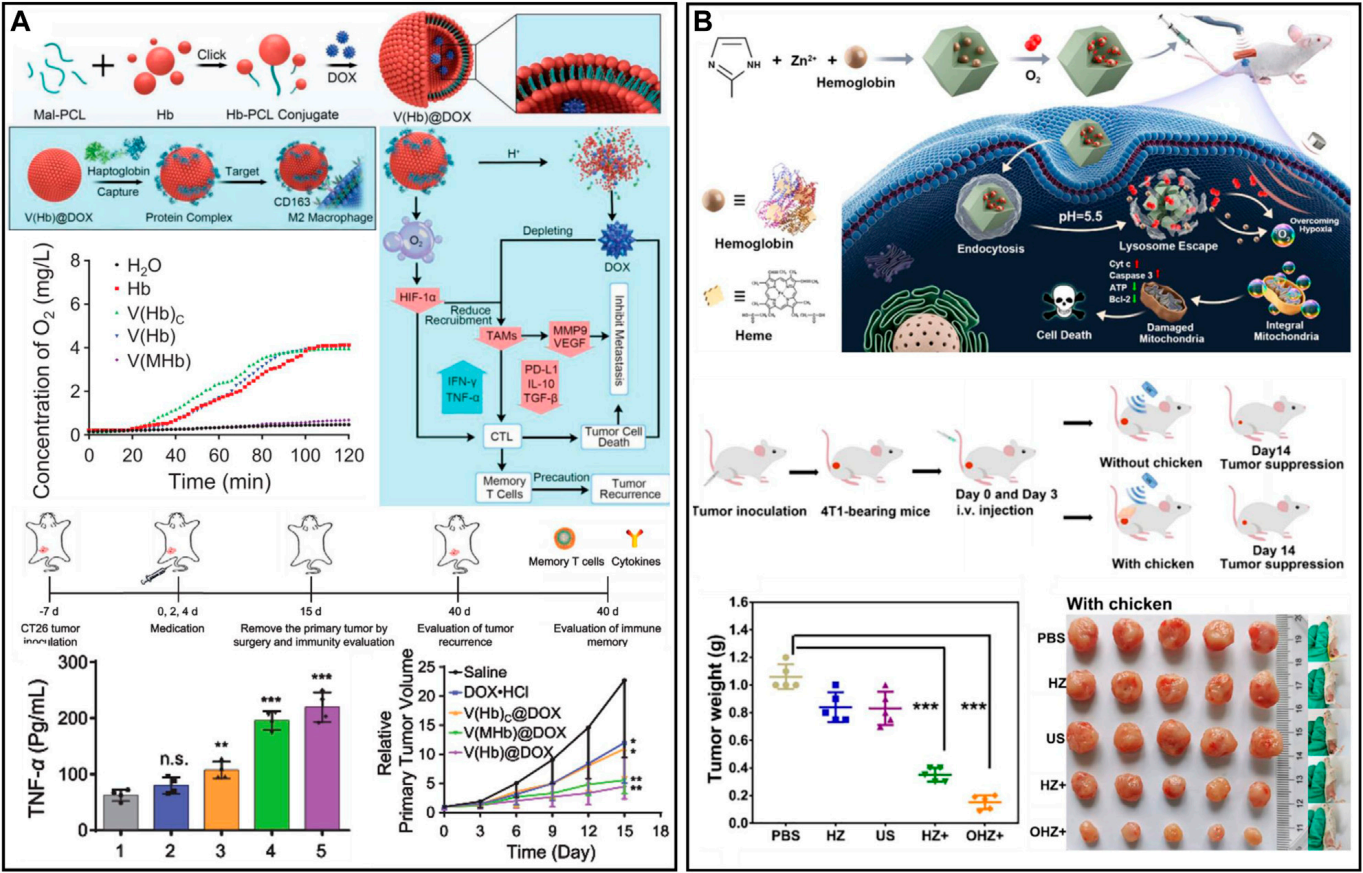
**(A)** V(Hb)@DOX enhanced cancer chemoimmunotherapy and reprogrammed the TME. Reproduced from ref. (Tian et al., 2017) with permission from Wiley, copyright 2021. **(B)** Nanomaterials combined with oxygen-carrying hemoglobin for sonodynamic therapy. Reproduced from ref. (Wang et al., 2021a) with permission from ACS Publications, copyright 2021.

Hemoglobin (Hb) is the core of red blood cell (RBC) binding and transporting oxygen, and oxygen forms a strong chemical covalent bond with the iron element in hemoglobin, which can reversibly bind oxygen molecules to form oxyhemoglobin (HbO_2_). Then, oxygen is provided to various tissues and organs *via* the circulatory system (Gell, 2018; Olson, 2022). However, the circulation time of free Hb is short, and its stability is poor, which limits its usage and application areas. To solve this problem, researchers have used various nanocarriers to load HbO_2_ by physical encapsulation to achieve the same oxygen-carrying function as RBCs (Jansman and Hosta-Rigau, 2018; Kim et al., 2023). A nanobionic oxygen carrier (DHCNPs) with the functions of targeting homologous cancer cells and increasing oxygen content was prepared by using polymer-loaded doxorubicin (DOX) and Hb as the core and coating of breast cancer cell membrane on the surface (Tian et al., 2017). The DHCNPs utilize adhesion molecules of cancer cells to target and identify homologous cancer cell tumors and combine with breast cancer cells, thereby realizing targeted administration to the same tumor while delivering chemotherapy drugs and oxygen to the interior of the tumor. The results showed that the hypoxic TME was altered by targeted oxygen replenishment. Therefore, the expression of HIF-1α and p-gp was reduced. The amount of DOX pumped out of the cancer cells was reduced, thereby improving chemotherapy resistance and realizing safe and efficient chemotherapy. However, the method of encapsulating Hb in nanocarriers requires the participation of organic solvents and severe stirring or ultrasonic operation, which might affect the activity of Hb. In addition, in the process of physical loading, to minimize the mutual repulsion between protein and protein, they are fixed in the form of random orientation, which is a non-uniform state that causes partial inactivation of the protein. Moreover, this high-density loading through intermolecular forces such as ionic hydrophobic and polar interactions will cause the blocking of protein active sites in space. To avoid this problem, Hb may be coupled to the carrier by covalent binding. Wang et al. (2021a) utilized Hb and polycaprolactone self-assembled nano erythrocyte systems to deliver DOX and oxygen [V(Hb)@DOX] to reprogram the tumor immunosuppressive microenvironment to enhance chemoimmunotherapy (Figure 2A). After administration, V(Hb)@DOX specifically targeted M2 macrophages with high CD163 expression in the tumor region. The oxygen released from V(Hb)@DOX alleviated tumor hypoxia, downregulated HIF-1α expression, and reduced the recruitment of M2-type macrophages. Since Hb is stable at neutral pH, it is partially dissociated in the acidic environment, resulting in the release of DOX to kill tumor cells. Ultimately, V(Hb)@DOX-mediated immune reprogramming could prevent tumor metastasis and recurrence. In addition, Hb can also be used as a sonosensitizer for cancer therapy (Yuan et al., 2021b) (Figure 2B). To improve the cellular uptake of Hb and protect it from hydrolysis by lysosomal enzymes, zeolite imidazolium 8 (ZIF-8) (composed of Zn^2+^ ions and 2-methylimidazole ligands) was used for drug encapsulation and controlled release. ZIF-8 was used as a drug carrier and could be taken up by cancer cells to achieve high Hb loading efficiency and pH-responsive O_2_ release from tumor sites. Oxygen release from Hb can enhance photodynamic behavior, which then induces severe mitochondrial dysfunction and activates the mitochondrial apoptotic pathway, further effectively inhibiting tumor cell growth. Compared with RBCs, the enhanced penetration and retention effects of Hb-based nanocarriers enabled them to accumulate more in hypoxic regions of tumors and improve oxygen delivery efficiency. Therefore, nano-oxygen carriers showed better oxygen supply capacity than natural erythrocytes in the TME.

#### 2.2.2 Perfluorocarbon-based oxygen nanocarriers

The solubility of O_2_ in perfluorocarbon is approximately 40–50 mL O_2_ per 100 mL; thus, it can be used as an oxygen carrier (Cheng et al., 2015; Wang et al., 2019; Jagers et al., 2021). In perfluorocarbons, oxygen undergoes physical dissolution, and there is a weak non-directional van der Waals force between oxygen and perfluorocarbons, which also ensures that oxygen can be dissolved or released quickly. Various perfluorocarbon (PFC)-based oxygen delivery nanoplatforms, such as liposomes, hollow nanomaterials or polymers, have been successfully fabricated and utilized to alleviate tumor hypoxia and enhance oxygen-related therapeutic efficacy (Cheng et al., 2015; Gao et al., 2017; Li et al., 2018; Liang et al., 2020; Dong et al., 2022). However, the premature release of oxygen before reaching the anoxic region and the oxygen transport mainly driven by the concentration gradient are not controllable. Therefore, the development of controllable oxygen carriers is significant for the delivery of oxygen (You et al., 2020b). In view of the weak van der Waals interactions between PFC and oxygen, ultrasound and near-infrared light (NIR) are currently the main stimuli to trigger oxygen release (Yang et al., 2019). For example, Song et al. (2016) used human serum albumin as a stabilizer for PFC nanoemulsions and selected ultrasound to induce oxygen release to improve the hypoxic environment of tumors. The nanoemulsion was injected into tumor-bearing mice. The PFC nanoemulsion absorbed oxygen from the lungs and circulated to the hypoxic tumor site. Another common source of excitation is the NIR laser. For example, a “nano bomb” (PFH@Ag@Ch-I) was designed using indocyanine green (ICG), chitosan, and perfluorohexane (PFH) (Yang et al., 2021). When PFH@Ag@Ch-I was endocytosed by lysosomes, the photothermal activity of the silver nanocages made PFH reach the boiling point and rapidly vaporize under NIR irradiation, which led to the release of oxygen. The increased internal pressure of the “nano bomb” resulted in silver nanocage explosion and enabled lysosome escape. Then, the silver nanocages were broken into small nanoparticles for a Fenton-like reaction, and the oxygen released from PFH improved the hypoxic TME and enhanced ICG-mediated photodynamic therapy (PDT), thereby achieving efficient tumor penetration and antitumour therapy. To increase the tissue penetration depth, Zhang et al. (2022d) encapsulated PFCs in functionalized polymers to prepare a photothermo-triggered “oxygen bomb.” By using an NIR-II laser, the combination of photothermal therapy (PTT), PDT, and chemotherapy could be released.

#### 2.2.3 Monocyte-based oxygen nanocarriers

Studies have found that several chemokines secreted by tumor cells are closely related to the recruitment of monocytes (Murdoch et al., 2004). Once monocytes reach the tumor site and further develop into tumor-associated macrophages (TAMs), they tend to be retained in hypoxic tumor tissue (Brown and Wilson, 2004). For example, bone marrow-derived monocytes were used to carry oxygen-loaded polymer bubbles, superparamagnetic iron oxide (SPOIN) and the photosensitizer chlorin e6 (Ce6) (Huang et al., 2015). The multifunctional oxygen bubble carrier (SCOPB-engulfed monocytes) was not toxic to cells without external stimulation. When irradiated with a laser (660 nm) and high-frequency magnetic field, the growth of prostate xenografts in nude mice was significantly inhibited by combined magnetothermal therapy and PDT. The histological results of the tumor sections showed that the effective therapeutic agent could relieve tumor hypoxia and then enhance the therapeutic effect of PDT.

Oxygen-carrying nanocarriers with advantages, disadvantages, and major highlights are summarized in Table 2. PFC carries more oxygen than Hb at the same concentration. As the release of oxygen in PFC is achieved by concentration gradient, while that in Hb is related to Hb oxy-deoxy conformational change, the oxygen release efficiency of PFC is higher than that of Hb, and controlled release can be realized under external stimulation.

**TABLE 2:** Oxygen-carrying nanocarriers with advantages, disadvantages, and major highlights.

Classification	Advantage	Disadvantage	Major highlights	Ref
Hemoglobin-based oxygen nanocarriers	Excellent biocompatibility and safety	Poor stability; unpredictable side effects; limited oxygen loading efficiency and release	Reversibly combine with oxygen	Yang et al. (2018), Ciaccio et al. (2022)
Perfluorocarbon-based oxygen nanocarriers	Excellent biocompatibility; high stability; high oxygen solubility	Premature oxygen leak; complicated synthesis process	Controlled release of oxygen under external stimulation	Cheng et al. (2015), Gao et al. (2017), Li et al. (2018), Liang et al. (2020), Dong et al. (2022)
Monocyte-based oxygen nanocarriers	Excellent biocompatibility	Limited oxygen loading efficiency; Complex extraction process	Easily remain in hypoxic tumors	Huang et al. (2015)

### 2.3 Oxygen-production nanomaterials

The content of hydrogen peroxide (H_2_O_2_) in tumor tissue is generally higher than that in normal tissues, which is one of the reasons for tumor invasion and metastasis. This characteristic also offers an opportunity for alleviating tumor hypoxia; for instance, catalase (CAT) can catalyze the decomposition of H_2_O_2_ to generate O_2_ *in situ*. With the development of nanotechnology, a variety of nanomaterials with catalytic properties have been discovered (Wei and Wang, 2013; Liang et al., 2019; Wang et al., 2022e). Among these nanozymes, some nanozymes with CAT-like activity have been developed. In this section, we will summarize nanomaterials combined with natural CAT enzymes and CAT-like nanozymes to enhance tumor therapy.

#### 2.3.1 CAT-loaded nanoagents

**FIGURE 3:**
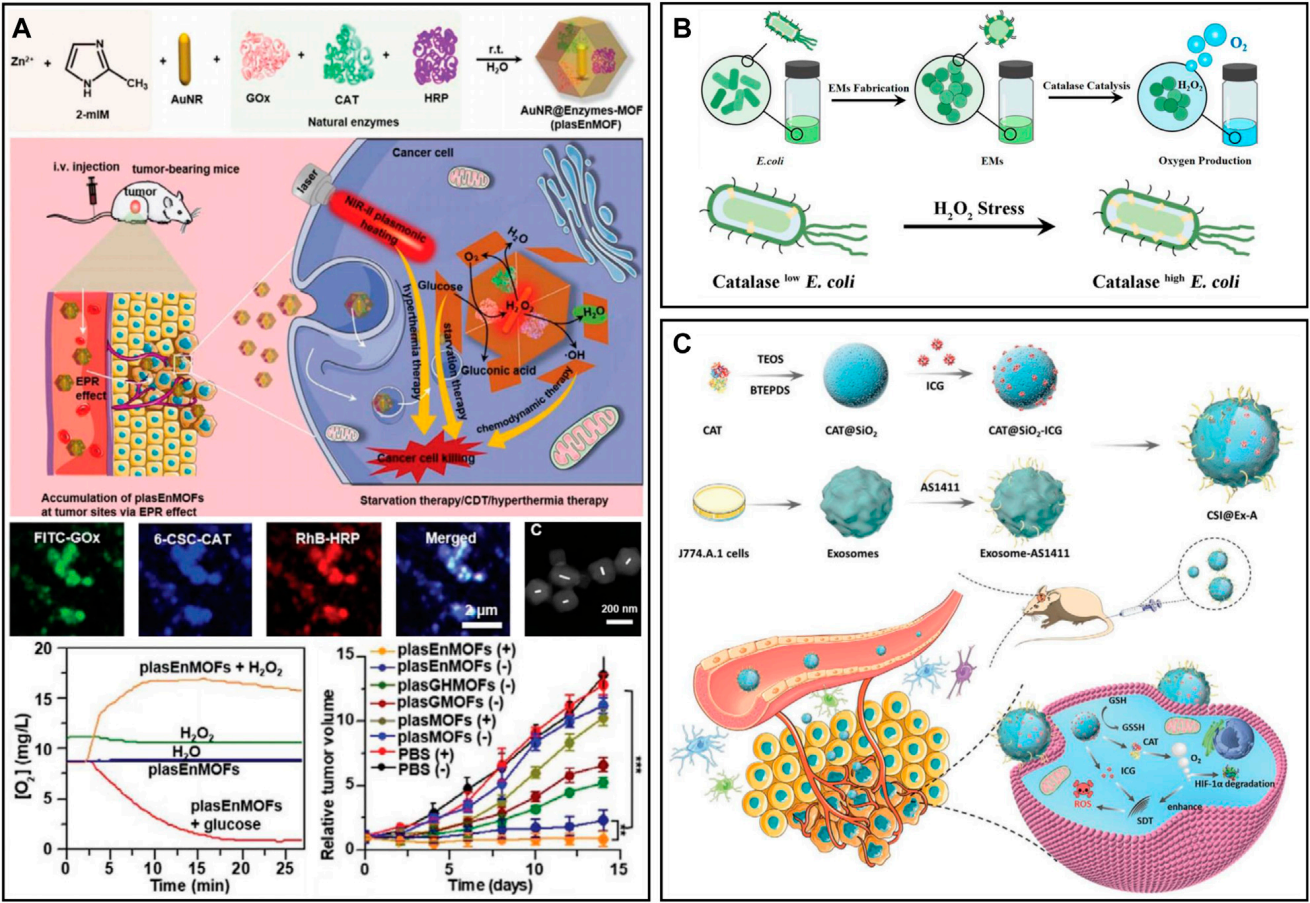
**(A)** PlasEnMOF for enzymatic cancer therapy. Reproduced from ref. 40 with permission from Wiley, copyright 2022. **(B)** EMs for tumor hypoxia relief. Reproduced from ref. (Huang et al., 2015) with permission from ACS Publications, copyright 2021. **(C)** CSI@Ex-A for O_2_-self-supply SDT. Reproduced from ref. (Qin et al., 2021) with permission from Wiley, copyright 2022.

CAT is a common natural enzyme in living organisms that can catalyze H_2_O_2_ to produce oxygen (Goyal and Basak, 2010; Ma et al., 2019; Mu et al., 2023). To exert an excellent cascading catalysis reaction, Zhou and Li (2022) designed a three-enzyme cascade nanosystem (plasEnMOF) by embedding CAT, glucose oxidase (GOx) and horseradish peroxidase (HRP) in ZIF-8-encapsulated gold nanorods (AuNRs) (Figure 3A). After intravenous injection of plasEnMOF in 4T1 tumor-bearing mice, the overexpressed H_2_O_2_ in tumors could be converted into oxygen by CAT, thereby relieving tumor hypoxia and providing the oxygen required for GOx to react with glucose. GOx utilized oxygen to catalyze glucose to produce H_2_O_2_, which could convert into hydroxyl radicals (•OH) for CDT in the presence of HRP. In another work, to significantly increase reactive oxygen species (ROS) levels in the TME, Qin et al. (2021) prepared nanogels (FIGs-LC) by integrating lactate oxidase (LOx) and CAT into hybrid nanogels that encapsulated with Fe_3_O_4_ NPs and ICG. LOx can catalyze endogenous lactic acid to produce H_2_O_2,_ which reacts with Fe_3_O_4_ NPs to produce •OH. Meanwhile, the oxygen generated from the CAT catalytic reaction could enhance singlet oxygen production. The *in vivo* results showed that the tumor inhibition rate of FIG-LC reached 89.05%, and the side effects were negligible. However, the catalytic efficiency of CAT may be affected by numerous proteases *in vivo*, especially in tumor sites where proteases are overexpressed (Vandooren et al., 2016; Zai et al., 2021). Recently, Zai et al. (2021) developed CAT-containing *Escherichia coli* membrane vesicles (EMs), which showed good protease resistance for alleviating tumor hypoxia and radiotherapy enhancement (Figure 3B). The CAT in EMs was more than 100 times more resistant to protease than free CAT and was able to maintain catalytic activity even 12 h after intratumoral injection. In a separate study, Wu et al. (2022) prepared a biodegradable nanoplatform (CSI@Ex-A) by loading CAT and ICG into silica nanoparticles and then coated it with AS1411 aptamer-modified macrophage exosomes (Figure 3C). After endocytosis of tumor cells, the highly expressed glutathione (GSH) triggered the degradation of CSI@Ex-A to release CAT, which could catalyze H_2_O_2_ to generate O_2_ to alleviate tumor hypoxia. Furthermore, the therapeutic effect of sonodynamic therapy (SDT) was enhanced by GSH depletion and O_2_ self-supply *in vitro* and *in vivo*.

#### 2.3.2 CAT-like nanozymes

##### 2.3.2.1 Au-based nanozymes

Due to their excellent photothermal properties in the near-infrared region, Au-based nanomaterials are often used as photothermal agents for PTT (Cheng et al., 2017; Ye et al., 2022). In recent years, it was found that Au-based nanomaterials have exhibited a variety of enzymatic activities, such as peroxidase, CAT, superoxide dismutase (SOD), and oxidase, among which CAT-like and SOD-like activities can be used to alleviate tumor hypoxia (Lin et al., 2014; Lou-Franco et al., 2021). pH has an important effect on the catalytic activity of AuNPs (He et al., 2013). In a neutral pH environment, AuNPs exhibit SOD-like activity that can convert superoxide into H_2_O_2_. In alkaline environments, AuNPs exhibit CAT-like activity that converts H_2_O_2_ into oxygen. However, in an acidic pH environment, the SOD-like and CAT-like activities of AuNPs are significantly reduced. To improve the catalytic activity of AuNPs in acidic pH to some extent, Yang and coworkers first reported amine-terminated polyamidoamine (PAMAM) dendritic molecule-encapsulated Au nanoclusters (AuNCs-NH_2_) (Liu et al., 2017a). The 3-amines on dendritic macromolecules were easily protonated in an acidic TME, which facilitated the preadsorption of OH^−^ on the gold nanoclusters. Furthermore, the CAT-like activity of the nanoclusters was extended to an acidic TME (pH 4.8–7.4), catalyzing the production of oxygen from overexpressed H_2_O_2_ to alleviate tumor hypoxia and enhance PDT. In a similar study, Xu et al. (2019) designed a multifunctional nanocomposite (PGPAI NPs), which was polypyrrole (PPy) coated by graphene oxide (GO) flakes and then modified with PEG, Au NPs and IR820 molecules. The PGPAI NPs had good photoacoustic imaging and computed tomography imaging capabilities. Under NIR irradiation, PPy and IR820 could effectively produce heat and ROS, respectively. Au NPs not only generate oxygen by catalyzing overexpressed H_2_O_2_ in the tumor, enhancing the effect of oxygen-dependent PDT but also exhibit GOx like activity and can efficiently catalyze the conversion of glucose into H_2_O_2_ and gluconic acid (Li et al., 2020c; Chen et al., 2021a). Another classic example was presented by Liu et al. (2020a). An iron-based metal organic framework (GIM) doped with AuNPs was used for ROS production in hypoxic tumors *via* cascade catalytic reactions (Liu et al., 2020a). Due to the GOx like activity of AuNPs, GIM could catalyze glucose to H_2_O_2_ and then generate •OH for CDT through the Fenton reaction. In addition, GIM could also rapidly decompose H_2_O_2_ into O_2_, which not only alleviated hypoxia in the TME but also promoted the catalysis of glucose by AuNPs.

##### 2.3.2.2 Manganese-based nanozymes

**FIGURE 4:**
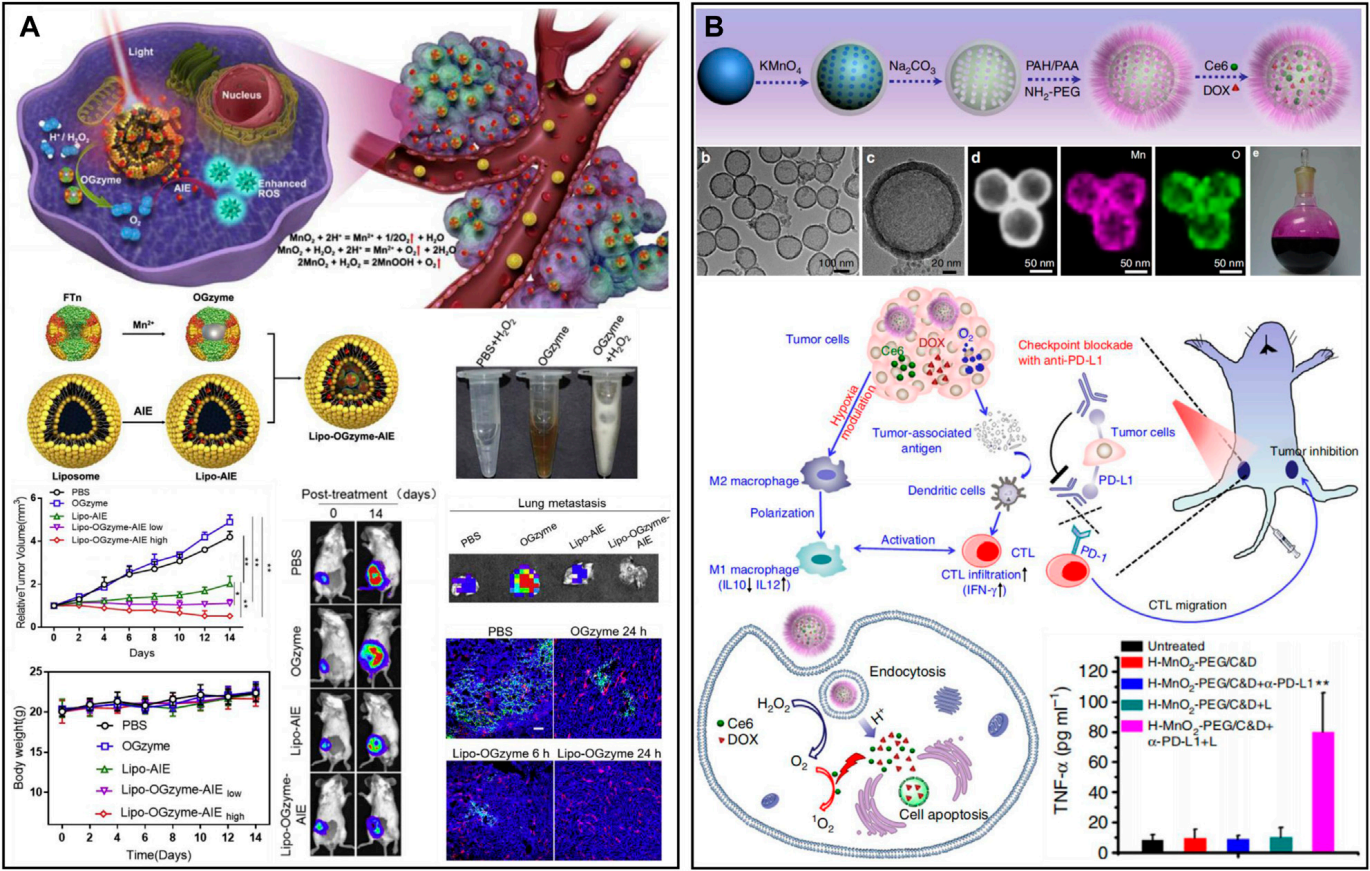
**(A)** A response cascade theranostic nanosystem (Lipo-OGzyme-AIE) for photodynamic therapy. Reproduced from ref. (Liu et al., 2020a) with permission from Elsevier, copyright 2019. **(B)** Hollow MnO_2_ nanotherapeutics for combination therapy. Reproduced from ref. (Zhou et al., 2020a) with permission from Springer Nature, copyright 2017.

Manganese dioxide (MnO_2_) can catalyze excessive H_2_O_2_ in the TME to produce oxygen, demonstrating unprecedented advantages in the treatment of TME hypoxia due to its good degradation ability as well as high catalytic activity (Ding et al., 2020; Gao et al., 2020). A responsive cascade theranostic nanosystem (Lipo-OGzyme-AIE) was designed by encapsulating aggregation-induced emission (AIE) and OGzymes in the phospholipid bilayer (Figure 4A) (Gao et al., 2020). The O_2_ generated through the catalytic reaction of MnO_2_ could diffuse throughout the tumor, providing O_2_ for AIE to produce singlet oxygen under irradiation. In addition, bovine serum albumin (BSA) was designed to encapsulate gold nanorods (Au NRs), and MnO_2_ nanoparticles were deposited at the reduction site to form Au NRs@BSA/MnO_2_ (Zhou et al., 2020a). Based on the strong localized surface plasmon resonance effect of Au NRs, this nanosystem had good photothermal conversion efficiency and could be used for photothermal ablation of tumors. The MnO_2_ particles from the nanosystem could decompose H_2_O_2_ to produce O_2_, which in turn could be used for hypoxia improvement in the TME. However, the previously reported structures of MnO_2_ are mostly nanoparticles, nanocomposites in combination with other types of nanoparticles or nanosheets, which may not be ideal for the effective loading and accurate release of drugs. Hollow nanostructures have been shown to be able to construct loading/delivery nanoplatforms for the precisely controlled release of therapeutic agents (Zhang et al., 2022f; Zhu et al., 2022). The research group of Liu developed a biodegradable hollow MnO_2_ nanotherapeutic (Figure 4B) (Yang et al., 2017). Nanotherapeutics can achieve TME-responsive imaging and specific release of drugs, improve the hypoxic environment of tumors and enhance the effect of cancer treatment. Finally, the nanotherapeutic could be rapidly decomposed in the mouse body and excreted out of the body. Based on this nanotherapeutics, the synergistic therapeutic effects of chemotherapy and PDT *in vivo* could be effectively improved. After nanotherapeutics are combined with checkpoint blockade, anti-programmed death ligand 1 (anti-PD-L1) therapy can not only kill the primary tumor but also effectively inhibit the growth of distant tumors. In addition to being a nanozyme, MnO_2_ can also react with H^+^ or GSH existing in the TME and decompose to Mn^2+^, which prominently enhances T1 magnetic resonance imaging contrast and can be used for tumor-specific imaging (Xiao et al., 2021; Li et al., 2022c; Pan et al., 2022).

##### 2.3.2.3 Platinum-based nanozymes

**FIGURE 5:**
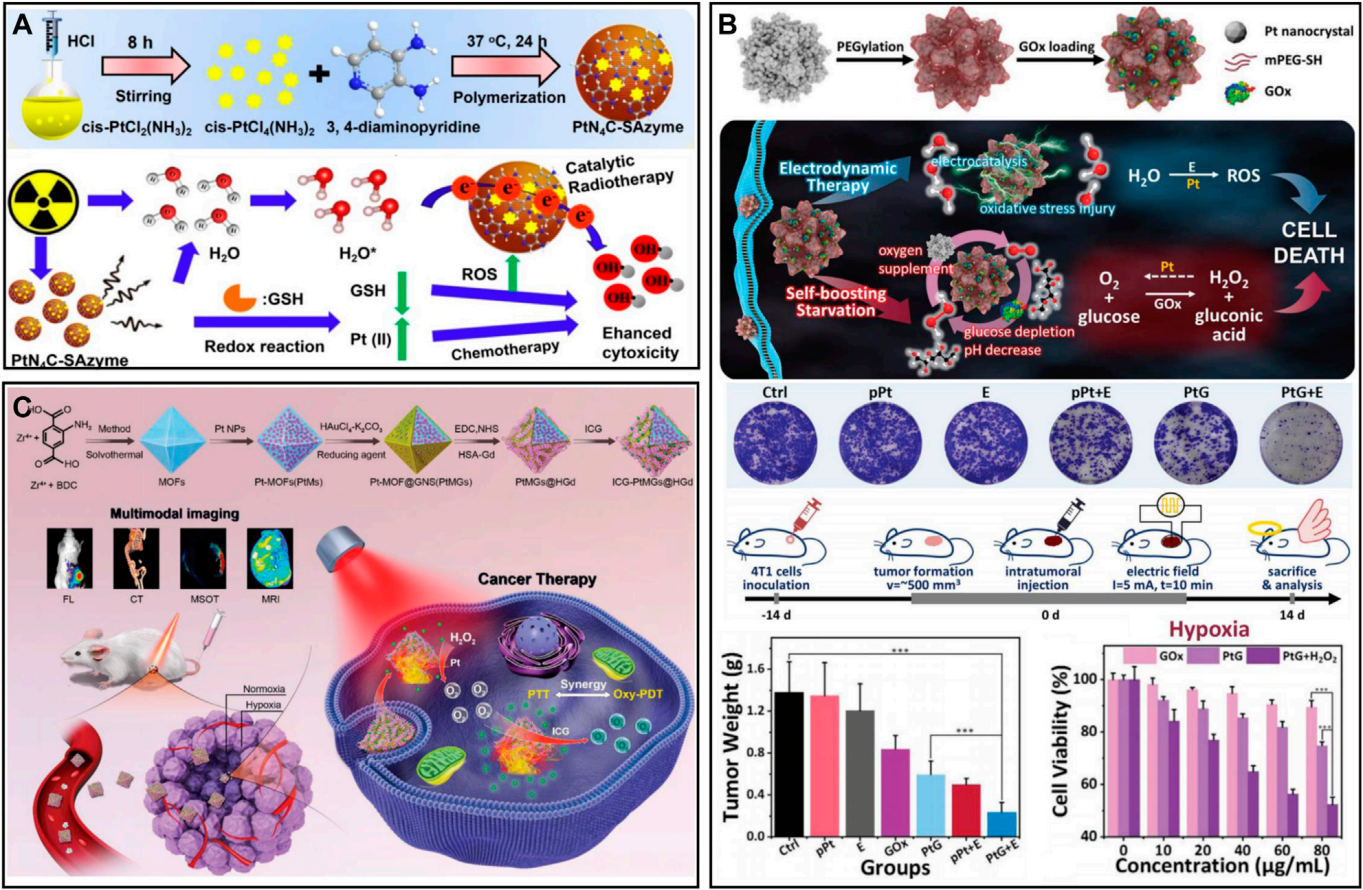
**(A)** PtN_4_C-SAzyme for oxygen self-supplied tumor therapy. Reproduced from ref. (Yang et al., 2017) with permission from Ivyspring International Publisher, copyright 2022. **(B)** Porous platinum nanospheres for tumor combination therapy. Reproduced from ref. (Xu et al., 2022b) with permission from Wiley, copyright 2020. **(C)** ICG-PtMGs@HGd for hypoxia relief and tumor therapy. Reproduced from ref. (Lu et al., 2020) with permission from Wiley, copyright 2020.

Platinum (Pt)-based nanomaterials are widely used as nanozymes for oxygen production due to their low production cost and long-lasting catalytic properties (Chen et al., 2021b). At present, the development of nanodrug responses to the TME is a common strategy to treat tumors. A TME-activated nanodrug (PtN_4_C-SAzyme) prepared by Xu et al. (2022b) was used for the cascade catalytic reaction (Figure 5A). SAzyme has peroxidase (POD)-like and CAT activities. The overexpressed GSH in cells was continuously consumed by Pt (IV) to generate Pt^2+^, which could reduce ROS scavenging. More importantly, PtN_4_C-SAzyme had SOD-like activity, which converted O_2_^−^ into H_2_O_2_ to supplement the consumption of H_2_O_2_, and H_2_O_2_ could also further react with PtN_4_C-SAzyme to realize the cycle of •OH and O_2_^−^. To improve the stability of Pt-based nanomaterials in aqueous solution, Lu et al. (2020) performed PEGylation of platinum porous nanospheres (pPts) and loaded GOx (Figure 5B). GOx could convert glucose to H_2_O_2_ in the presence of oxygen; pPts subsequently decomposed the overexpressed H_2_O_2_ in tumors into oxygen and water. Thus, pPts promoted the consumption of glucose in hypoxic tumors and increased cellular oxidative stress. In addition, Pt assisted by a direct current electric field and chloride ions induced the decomposition of water molecules on their surface, generating cytotoxic •OH. pPts-mediated electrokinetic therapy in synergy with starvation therapy could significantly alleviate the hypoxic microenvironment and inhibit tumor growth. Interestingly, You et al. (2020a) developed a nanomaterial (ICG-PtMGs@HGd) that could continuously replenish O_2_ with low toxicity (Figure 5C). Pt and Au successively wrapped metal-organic frameworks (MOFs) to form octahedral metal nanoshells (PtMGs), and then the surface was modified with gadolinium-chelated human serum albumin (HSA-Gd) and ICG. Oxygen production was measured with a dissolved oxygen meter. After H_2_O_2_ addition, the oxygen concentration varied from 5.0 to 10.8 mgL^−1^ in the ICG-PtMGs@HGd solution without significant oxygen production in the absence of ICG-MGs@HGd, and this catalytic effect was durable. In the synergistic phototherapy experiment *in vivo*, the results showed that the tumor volume of the ICG-PtMGs@HGd group under NIR irradiation decreased most significantly, and the body weight of the tumor-bearing mice did not change significantly after treatment. In general, there is a wide range of biological applications of Pt-based nanomaterials, but the toxic side effects caused by heavy metals are still current challenges to be overcome (Duan et al., 2022; Velcheva et al., 2022).

##### 2.3.2.4 Other metal-based nanozymes

**FIGURE 6:**
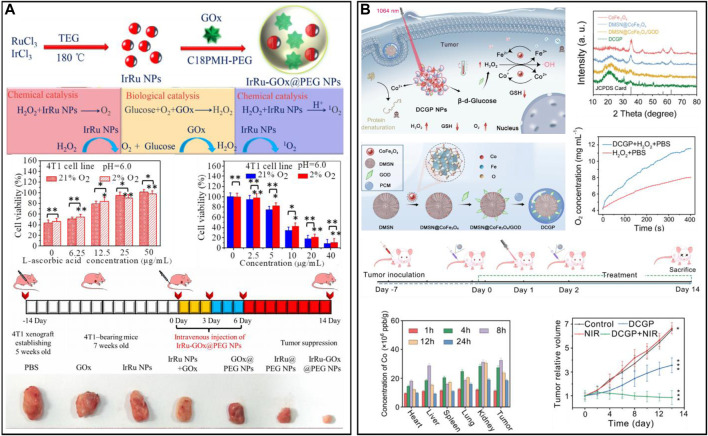
**(A)** A multienzyme nanoreactor (IrRu-GOx@PEG NPs) for enhanced oxidation therapy. Reproduced from ref. (You et al., 2020a) with permission from Elsevier, copyright 2020. **(B)** DMSN@CoFe_2_O_4_/GOD-PCM for reshaping the tumor microenvironment. Reproduced from ref. (Wei et al., 2020) with permission from ACS Publications, copyright 2022.

With the action of oxygen and GOx, glucose can be converted into gluconic acid and H_2_O_2_, thereby cutting off the nutrition source of tumor cells and inhibiting cancer cell proliferation (Huo et al., 2017; Xu et al., 2022c; Wang et al., 2022f). Considering that the catalytic activity of a single enzyme is insufficient to achieve satisfactory therapeutic effects, the development of nanozymes with multiple enzyme-mimetic functions is necessary. A multienzyme nanoreactor (IrRu-GOx@PEG NPs) for cascade catalytic reactions was prepared after the rRu alloy nanoparticles were modified with GOx and PEG (Figure 6A) (Wei et al., 2020). In the biological catalytic stage, the glucose in the tumor was degraded to H_2_O_2_ by IrRu-GOx@PEG NPs, which cut off the tumor’s nutrient source and inhibited tumor growth. In the chemical catalytic stage, IrRu-GOx@PEG NPs catalyzed H_2_O_2_ to generate O_2_ and highly toxic singlet oxygen (^1^O_2_). The *in vitro* and *in vivo* results indicated that the IrRu-GOx@PEG NPs could effectively induce 4T1 cell apoptosis. In addition, a nanoplatform (DMSN@CoFe_2_O_4_/GOD-PCM) was designed for NIR II-enhanced tumor therapy by depositing ultrasmall cobalt ferrite (CoFe_2_O_4_) and GOx into the pore size of dendritic mesoporous silica (Chang et al., 2022) (Figure 6B). After laser irradiation, the high temperature generated by CoFe_2_O_4_ melts the phase change material (PCM) to release GOx, remodelling the TME through the glucose metabolism pathway. The resulting intensified acidic conditions and large amount of H_2_O_2_ effectively initiate the cascade catalytic reaction. To achieve precise treatment, an upconversion nanoparticle (UCNP)-based smart nanosystem (UCNPs@Cu-Cys-GOx) was designed for cancer combination therapy (Wang et al., 2022a). The nanosystem remained inert (turned off) in normal tissues and was only specifically activated (turned on) in the TME through a sequence of enzymatic cascades. Moreover, the enhanced oxidative stress of the nanosystem could reverse the immunosuppressive TME. Meanwhile, the smart nanosystem combined with immunotherapy/starvation/chemokinetic synergistic therapy effectively inhibited primary tumor growth and cancer metastasis. In addition, GOx-induced starvation therapy synergized with copper death and significantly inhibited tumor growth (Fu et al., 2018; Xu et al., 2022d).

##### 2.3.2.5 Prussian blue-based nanozymes

**FIGURE 7:**
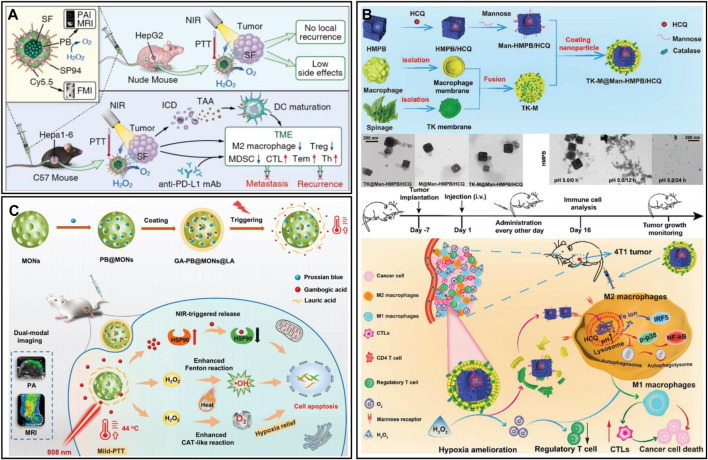
**(A)** SP94-PB-SF-Cy5.5 for tumor photothermal therapy and immunotherapy. Reproduced from ref. (Wang et al., 2022a) with permission from ACS Publications, copyright 2020. **(B)** GA-PB@MON@LA for synergistic photothermal and nanocatalytic therapy. Reproduced from ref. (Zhou et al., 2020b) with permission from Wiley, copyright 2022. **(C)** TK-M@Man-HMPB/HCQ for macrophage polarization and hypoxia relief. Reproduced from ref. (Tian et al., 2022) with permission from Wiley, copyright 2022.

Prussian blue (PB) is a Food and Drug Administration-approved antidote for heavy metal poisoning (Wang and Cheng, 2020). In recent years, researchers have gradually found that PB has excellent physicochemical properties, such as photothermal conversion and catalytic activity (Liu et al., 2016b; Wang and Cheng, 2020). PB can catalyze the production of oxygen from overexpressed H_2_O_2_, which can effectively improve the malignant environment (Komkova and Karyakin, 2022). Motivated by this, Zhou et al. (2020b) designed a PB nanoplatform (SP94-PB-SF-Cy5.5 NPs) loaded with sorafenib (SF) and modified with the hepatocellular carcinoma (HCC)-specific targeting peptide SP94 and the near-infrared cyanamide dye (Cy 5.5) (Figure 7A). In the treatment of HCC, the combination of PTT and SF could effectively reduce local tumor recurrence and the side effects caused by drugs. In addition, the POD-like activity and photothermal effect of PB could effectively reshape the hypoxic and immunosuppressive TME. When combined with anti-programmed death ligand 1 (PD-L1), immunotherapy can promote dendritic cell (DC) maturation and increase tumor infiltration of cytotoxic T lymphocytes (CTLs). More importantly, combination therapy could establish long-term immune memory and inhibit tumor metastasis and recurrence. Excess H_2_O_2_ in tumors can not only be used as a raw material for oxygen generation but also generate highly toxic ROS through the Fenton reaction, which can effectively kill tumor cells in combination with PTT. Tian et al. (2022) obtained an NIR-responsive therapeutic nanoplatform (GA-PB@MON@LA) by sequentially introducing PB and gambogic acid (GA) into the pores of mesoporous organosilicon (MONs) and coating them with the thermosensitive material lauric acid (LA) (Figure 7B). Under NIR laser irradiation, the nanoplatform could not only induce tumor cell apoptosis by PTT but also shed LA coatings, thus facilitating the release of GA. GA inhibited the expression of HSP90 and further suppressed tumor heat resistance. In addition, the heat generated by PTT could enhance the CAT-like and Fenton-like catalytic activities of PB, promoting the production of oxygen and •OH. The *in vitro* and *in vivo* experimental results showed that GA-PB@MON@LA has good antitumour effects and can be used as a PA/MR dual-modality imaging contrast agent to provide precise guidance for cancer treatment. To minimize the effect of reticuloendothelial system clearance, nanomaterial biomineralization is an effective strategy. Cytomembranes have homology targeting and good biocompatibility, which can protect nanomaterials from immune recognition of the body to prolong the blood circulation time and increase their enrichment at the focal site (Malaviya et al., 2019; Briolay et al., 2021). Hou et al. (2022) obtained hollow mesoporous PB nanoplatforms (TK-M@Man-HMPB/HCQ) by loading hydroxychloroquine (HCQ), modifying mannose and coating macrophage and thylakoid (TK) membranes (Figure 7C). With the homing action of macrophage membranes, the nanoplatform was able to achieve enrichment in tumor tissue. The TK membrane then catalyzed the generation of oxygen from high concentrations of H_2_O_2_ in the TME. During the process, the generation of oxygen promoted the rupture of the hybridized membrane, exposing Man-HMPB/HCQ. Man-HMPB/HCQ significantly enhanced macrophage internalization and induced polarization of M2 macrophages toward the M1 phenotype. *In vivo* results showed that the nanoplatform significantly inhibited tumor growth through a series of responses, including TAM polarization, CTL infiltration, alleviation of hypoxia and reduction in regulatory T cells.

#### 2.3.3 Nanomaterials with self-oxygen production performance

##### 2.3.3.1 Photocatalytic oxygen-producing nanomaterials

**FIGURE 8:**
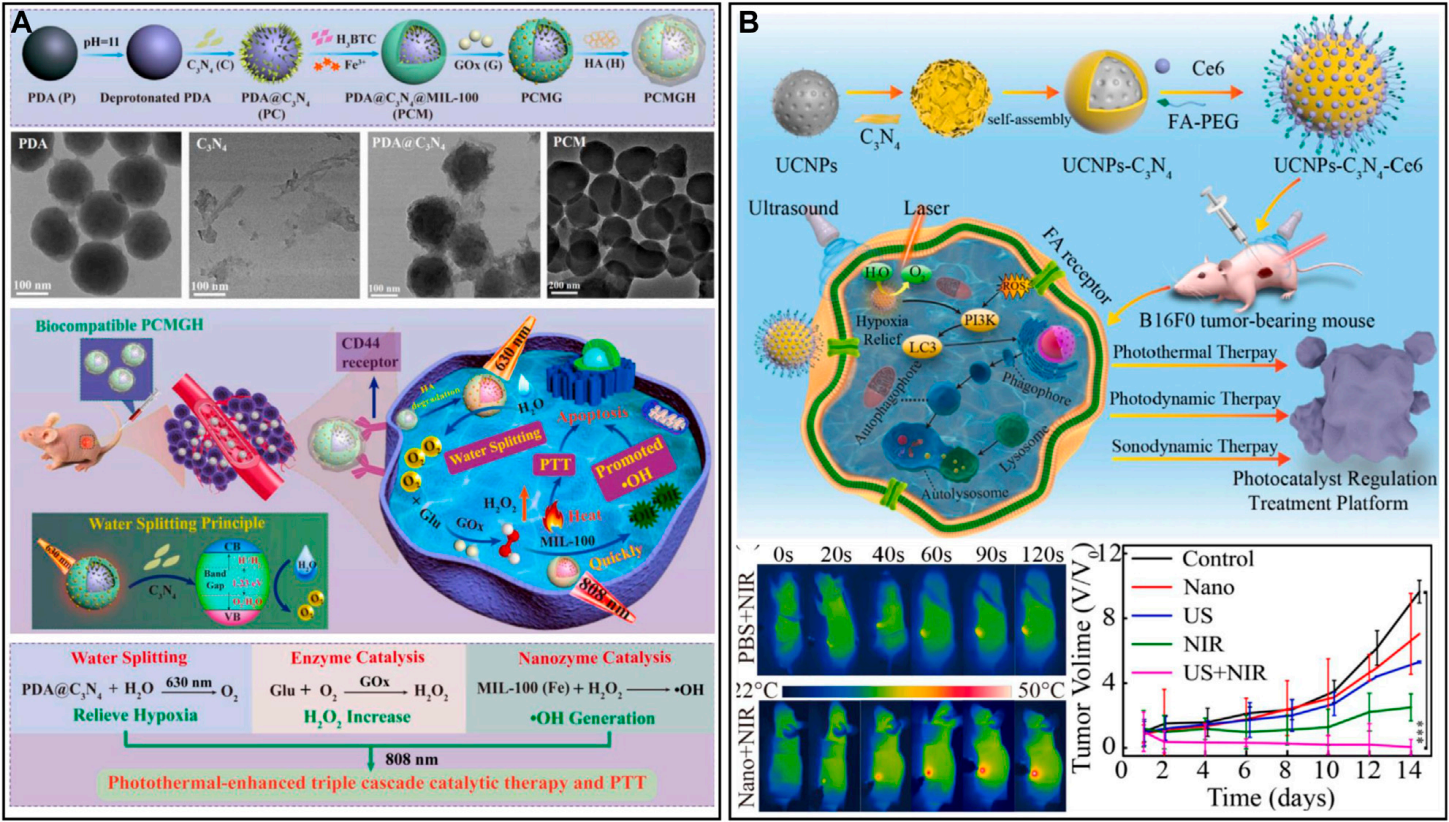
**(A)** Cascade catalyst PCMGH for tumor hypoxia therapy. Reproduced from ref. (Hou et al., 2022) with permission from Elsevier, copyright 2021. **(B)** UCNPs-C_3_N_4_-Ce6 for responsive tumor therapy. Reproduced from ref. (Wei et al., 2021) with permission from Elsevier, copyright 2021.

Photocatalytic water splitting is a clean, simple and sustainable pathway for producing O_2_ and hydrogen, which has led to great achievements in the fields of the environment, energy and biomedicine (Liao et al., 2020; An et al., 2021; Teng et al., 2021). Carbon nitride (C_3_N_4_) has been applied as a non-metallic photocatalyst for the photolysis of water in tumors to produce oxygen due to its visible light response and non-toxicity (Liu et al., 2020b; Ma et al., 2022). This endogenous oxygen production can enhance aerobic-based therapy. A cascade catalyst (PCMGH) was designed and obtained by assembling dopamine with C_3_N_4_ nanosheets, coating the surface with an iron-based metal-organic framework [MIL-100(Fe)], loading GOx, and grafting hyaluronic acid (Figure 8A) (Yu et al., 2022). A 630 nm laser could activate C_3_N_4_-mediated water splitting to generate oxygen in the tumor. Sufficient O_2_ further promoted GOx to consume endogenous glucose and generate the byproduct H_2_O_2_, and finally, MIL-100(Fe) with Fenton-like activity catalyzed H_2_O_2_ to generate •OH. During the cascade reaction, 808 nm NIR could elevate the reaction temperature of the tumor and enhance the catalytic performance to obtain more •OH. The efficient targeting ability of hyaluronic acid and the tumor environment response mechanism enable cascade catalysts to have both excellent biosafety and tumor efficacy. However, the disadvantages of C_3_N_4_ in complex physiological environments, such as the rapid recombination of electron-hole pairs and weak light absorption properties, limit its further application in biomedicine (Chen et al., 2022c). In this regard, researchers have designed ingenious modification methods to enhance the photocatalytic ability of C_3_N_4_ and effectively alleviate the endogenous hypoxia of tumors. Wei et al. (2021) designed a composite nanocatalyst by incorporating ruthenium (II) polypyridine complexes [Ru (bpy)_2_]^2+^ into graphitic C_3_N_4_ (gC_3_N_4_) *via* Ru-N bonds. The low-power visible light-induced separation and reduction of Ru_-_gC_3_N_4_ enhanced the therapeutic performance and biocompatibility of the composite catalysts in physiological environments. Mice were irradiated with 450 nm light (65 mW/cm^2^), which could generate oxygen and various ROS (•OH, •O_2_^−^ and ^1^O_2_) *in situ* in the tumor and greatly reduce the expression of HIF-1α protein. In another study, Zhang et al. (2022e) synthesized a heterojunction (UCNPs-C_3_N_4_-Ce6) by wrapping UCNPs with C_3_N_4_ and modifying with Ce6 and COOH-FA-PEG, which prevented the recombination of C_3_N_4_ electron-hole pairs, thereby enhancing the photoelectric conversion efficiency of nanomaterials (Figure 8B). UCNPs-C_3_N_4_-Ce6 with specific recognition ability increased tumor enrichment, and ultrasound (US) and NIR simultaneously stimulated the heterojunction to generate oxygen and ROS at the C_3_N_4_ interface, realizing the combined treatment of PDT, SDT, and PTT. The results of *in vivo* experiments showed that US + NIR-stimulated UCNPs-C_3_N_4_-Ce6 significantly upregulated the level of LC-3 protein in tumors, activated the autophagy pathway, and had good tumor elimination effects. Overall, heterostructure construction and elemental doping can improve the stability and photocatalytic activity of C_3_N_4_ *in vivo*, which is expected to modulate and remodel the hypoxic TME and reduce tumor metastasis and recurrence (Luo et al., 2018; Chen et al., 2021c).

##### 2.3.3.2 Microalgae-based oxygen generator

**FIGURE 9:**
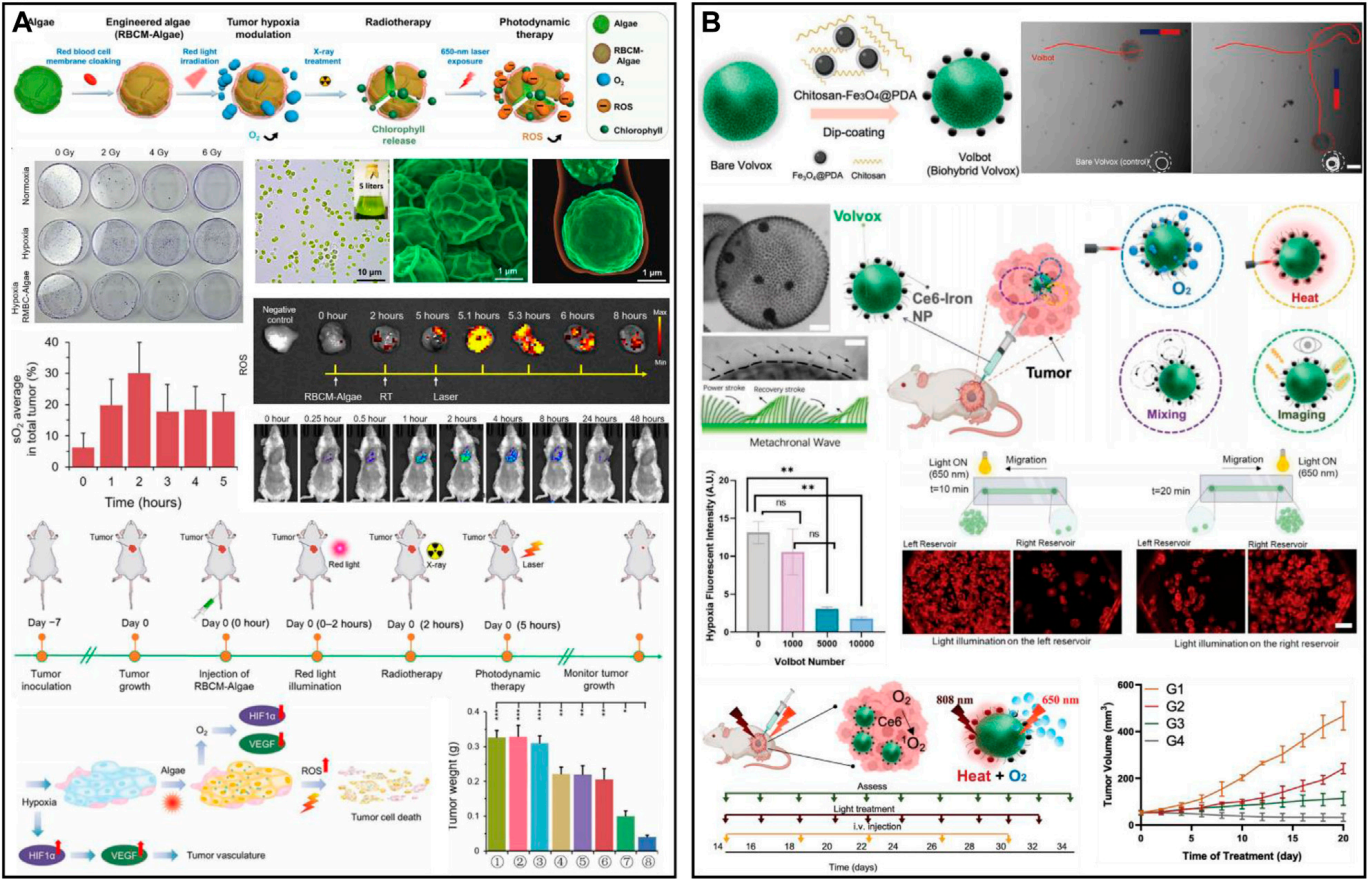
**(A)** RBCM-Algae for hypoxic cancer. Reproduced from ref. (Wang et al., 2022b) with permission from American Association for the Advancement of Science, copyright 2020. **(B)** Multifunctional microrobotic Volbot for tumor imaging and therapy. Reproduced from ref. (Qiao et al., 2020) with permission from Wiley, copyright 2022.

Microalgae produce oxygen by their specific photosynthesis, which has been applied for efficient oxygen production in tumors *in situ* to alleviate hypoxia and enhance oxygen-dependent therapies such as radiation therapy (RT) and PDT (Li et al., 2020d; Chen et al., 2021d; Zhong et al., 2021). Wang et al. (2022b) designed photosynthetic microcapsules (PMCs) by encapsulating UCNPs and *cyanobacteria* in an alginate microcapsule. The system achieved long-term oxygen supply by photosynthesis of cyanobacteria. PMCs could inhibit the NF-kβ pathway and downregulate the expression of HIF-1α, which created a hyperoxic microenvironment *in vivo* and significantly inhibited tumor growth and metastasis in hepatocellular carcinoma and mammary tumors. PMCs combined with checkpoint inhibitors (anti-PD-1) showed a powerful synergistic effect in mice with breast cancer. PMCs were intratumorally injected and had high tumor penetration. However, intravenously injected microalgae nanosystems are easily captured by the mononuclear phagocytic system (MPS) of the liver and spleen. To address the targeting problem, Qiao *et al.* developed erythrocyte membrane-encapsulated chlorella (RBCM-Algae) to reduce uptake and systemic clearance (Figure 9A) (Qiao et al., 2020). RBCM-Algae was delivered to tumors to produce oxygen *in situ* under red light-induced photosynthesis and improve RT. The 650 nm laser could release chlorophyll from microalgae, which further enhanced the ability to kill cancer cells by generating ROS through PDT. The rational combination of Fe_3_O_4_ NPs can further improve the precise delivery of microalgae to tumor tissues. Wang et al. (2022c) obtained an intelligent robot (Volbot) by combining volvox algae with Ce6-polydopamine@Fe_3_O_4_ through electrostatic interactions to achieve multimode imaging and oxygen generation (Figure 9B). Under the control of a magnetic field, the Volbot could move on a planned route. Red light (650 nm) irradiation could enhance the motion behavior of the Volbot and boost the mixing of biological fluids, facilitate the production of oxygen and improve the effect of PDT. In addition, Volbot could absorb NIR laser to generate localized thermotherapy to treat tumors. Volbots with magnetic resonance, photoacoustic, and fluorescence multimodal imaging offer great potential for achieving precise tumor treatment. In another work, Zhong et al. (2020) prepared tumor-targeted biohybrid microswimmers (PBNs) by combining *Spirulina platensis* with magnetic Fe_3_O_4_ NPs. The PBNs could be enriched in tumors using the guidance of magnetic fields, and oxygen production through photosynthesis could effectively alleviate hypoxia and improve the effectiveness of RT. In addition, chlorophyll can also generate ROS to enable PDT under laser irradiation. However, the application of microalgae in biomedicine is still in the preliminary stage, and some key issues remain to be solved, such as a laser selected for activating photosynthesis, microalgae size and morphology, targeting ability, biosafety, etc. (Hu et al., 2020; Wang et al., 2021b).

Thylakoids, distributed in the chloroplast stroma and cyanobacteria cells, are small flat capsules surrounded by a single-layer membrane. The thylakoid membrane contains photosynthetic pigments and electron transport chain components, which can convert light energy into chemical energy (Zheng et al., 2018; Cheng et al., 2021). For example, Zheng et al. (2018) constructed a phototriggered non-biological/biological thylakoid nanooxygen delivery system (PLANT) by coating the thylakoid membrane on the surface of synthetic nanoparticles, such as ZnONPs, Ag NPs, or SiO_2_ NPs. The delivery system can efficiently generate oxygen in the tumor under 660 nm laser irradiation, and *in vivo* and *in vitro* results showed that PLANT could reverse tumor hypoxia, inhibit anaerobic respiration, recover the metabolism of tumor cells to normal, regulate the abnormal structure and function of tumor blood vessels, limit the migration of tumor cells and remarkably improve the curative effect of PDT or antiangiogenesis treatment. *In vivo* results showed that, after treatment with PLANT, the levels of glycolysis-related enzymes (e.g., GLUT-1) in tumors were downregulated, and the CD31 protein and HIF-α were also significantly reduced.

##### 2.3.3.3 Metal peroxide-based oxygen generator

**FIGURE 10:**
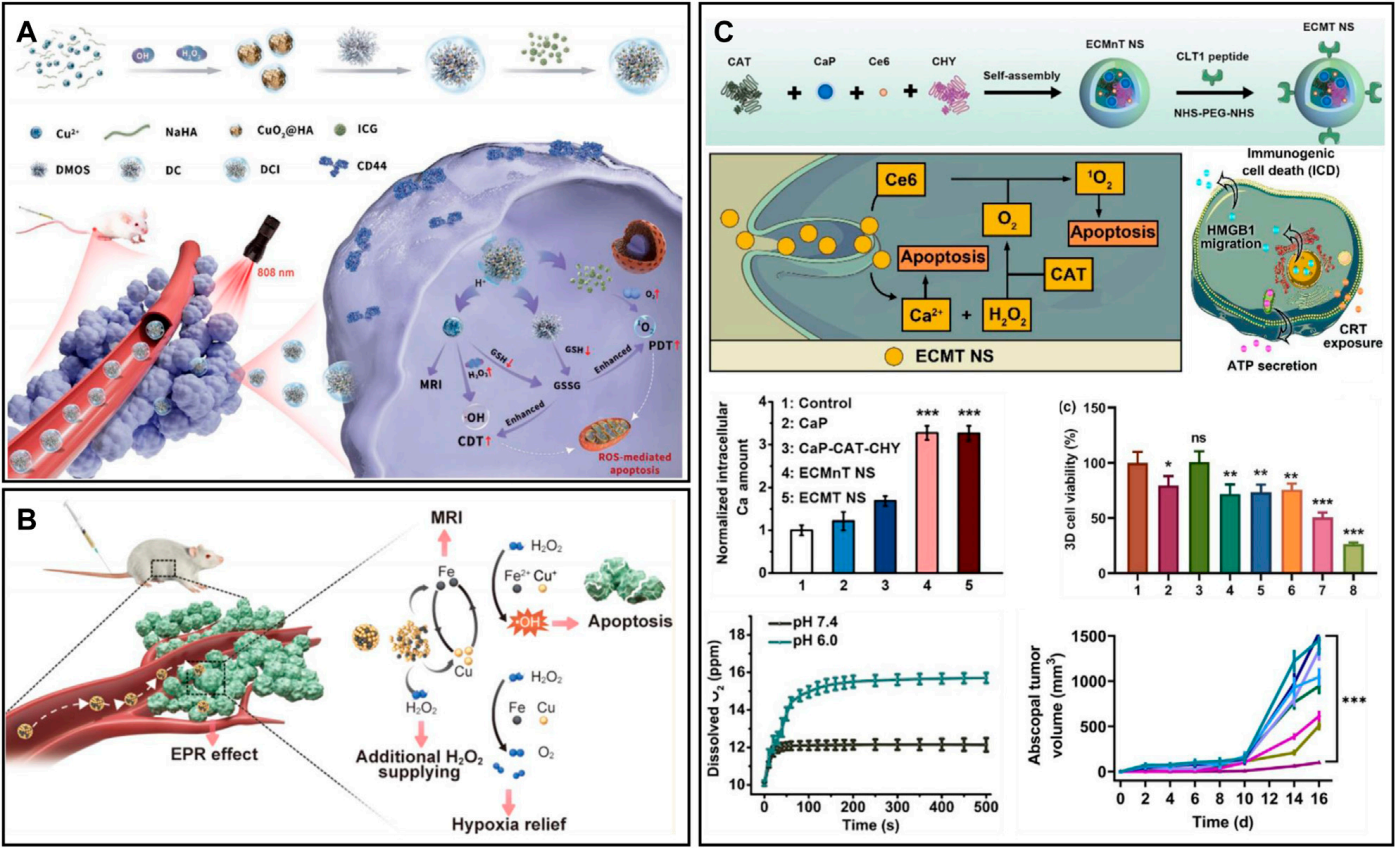
**(A)** DMOS@CuO_2_/ICG-HA for synergistic therapy. Reproduced from ref. (Zheng et al., 2018) with permission from Wiley, copyright 2022. **(B)** Copper−iron peroxide nanoparticles for the Fenton reaction. Reproduced from ref. (Wang et al., 2022d) with permission from ACS Publications, copyright 2022. **(C)** ECMT NS for tumor matrix destruction and cocktail therapy. Reproduced from ref. (Koo et al., 2022) with permission from Elsevier, copyright 2022.

Metal peroxides can decompose to produce H_2_O_2_ and metal ions under the acidic TME (Zhou et al., 2021). H_2_O_2_ has a wide range of applications in tumor therapy, such as increasing oxidative stress, providing oxygen, and providing catalytic substrate (Fu et al., 2018; Fu et al., 2021). Motivated by this, a combined nanotherapeutic (DCI) was synthesized by coloading CuO_2_ and ICG with dendritic mesoporous organosilica (DMOS) as a carrier and modifying with hyaluronic acid (Figure 10A) (Wang et al., 2022d). DCI can produce oxygen and H_2_O_2_ under acidic conditions to alleviate hypoxia. The ^1^O_2_ produced by NIR photoexcitation of ICG and the •OH produced by Cu^2+^-mediated Fenton-like reaction lead to the death of tumor cells. However, the limited efficiency of the Fenton-like reaction greatly affects metal peroxides for chemodynamic therapy (Wang et al., 2020b). Therefore, developing multicomponent catalytic metal composites through synergistic catalytic effects can achieve more satisfying catalytic performance (Zhou et al., 2021). Koo et al. (2022) reported a nanomaterial composite (CFp NPs) based on Cu-Fe perovskite nanoparticles (Figure 10B). The results showed that the CFp NPs synthesized with a Cu/Fe ratio of 7:3 had the best catalytic performance and could successfully ablate tumors at a low dose of 3.7 mg/kg. At the same time, CFp NPs alleviated tumor hypoxia by TME-responsive oxygen generation ability. The responsive release of ferric ions could also enhance T1-weighted MRI, enabling *in vivo* monitoring of tumors. Furthermore, metal ions dissociated by metal peroxides are also able to enhance the efficacy of tumor therapy. For example, Ca^2+^ released from CaO_2_ could induce calcium overload and cause mitochondrial damage (Liu et al., 2022); Ba^2+^ produced by BaO_2_ could act as a potassium ion channel inhibitor and inhibit tumor cell proliferation (Zhang et al., 2019). In addition, Chen et al. (2022a) designed a multifunctional nanoscavenger (ECMT NSs) by self-assembly of CAT, the digestive enzymes chymotrypsin (CHY), calcium peroxide nanoparticles (CaP), and the photosensitizer Ce6 and modified it with fibronectin-targeting CLT1 peptide (Figure 10C). Upon reaching solid tumors, the synergistic effect of CHY and ROS could effectively destroy the tumor matrix and facilitate the penetration of ECMT NSs. CaP could generate large amounts of Ca^2+^ and H_2_O_2_ in the acidic TME, which facilitated calcium ion therapy. Meanwhile, the generated H_2_O_2_ could be converted to oxygen in the presence of CAT, thus favoring the remodelling of the tumor hypoxic environment. The cooperation of calcium ion therapy and PDT could promote apoptosis and immunogenic cell death of tumor cells, which induce the activation of CTLs, thus reversing the immunosuppressive environment.

## 3 Conclusion and future perspectives

Hypoxia is one of the characteristics of the TME, and there is no linear relationship between tumor size and the degree of hypoxia, which may be present even if the tumor diameter is less than 1 mm (Li et al., 2007). Hypoxia leads to high invasion and metastasis of cancer cells, further making matters worse. In this paper, we review the advances in nanomaterial-based approaches to enhance the efficacy of various oxygen-related antitumour therapies by improving the TME by oxygen delivery and generation. Nanomaterials have achieved desirable results in enhancing tumor therapy. In clinical trials of hypoxic tumor treatment, a variety of personalized drugs have been developed (Table 3). NVX-108, the oxygen carrier of the second generation PFC, exhibits significantly higher oxygen delivery capacity, lower recommended dose, and fewer side effects than Flusol-DA with respect to direct oxygen delivery. At the same time, polyhedral hemoglobin has been further explored and practiced in clinical practice. Currently, a variety of drugs targeting hypoxia-related signaling pathways have been approved, but the results of various clinical trials are far from being as expected. Therefore, many critical issues must be carefully elaborated.

**TABLE 3:** Clinical trials of agents for hypoxia modulation in tumors.

Target	Agents	Agent type	Phase	Indication	Ref	
Oxygen supply	HBOC201	Polymerized Hb	II/III	Orthopedics/cardiac/thymoma	Mackenzie et al. (2019), Rubinstein et al. (2020)
	Fluosol-DA	PFC-based oxygen carrier	I/II	Glioblastoma; head and neck carcinoma	Rose et al. (1986)
	NVX-108	PFC-based oxygen carrier	I/II	Glioblastoma	Unger et al. (2017)
Hypoxia-ralated signaling pathways	2ME2 NCD	HIF-1α inhibitor	II	Myeloma; ovarian cancer; resistant prostate cancer	Matei et al. (2009)
	17-AGG	HIF-1α inhibitor	II	Metastatic renal cell carcinoma; relapsed lymphoma	Solit et al. (2008), Oki et al. (2012)
	Vorinostat	HIF-1α inhibitor	II/III	Gliobastoma; melanoma; lymphoma	Haas et al. (2014)
	Bevacizumab	VEGF antibody	Approval	Non-small cell lung cancer; colorectal cancer; breast cancer	Chen and Hurwitz (2018)
	Sunitinib	VEGFR inhibitor	III/Approval	Metastatic renal cell carcinoma (Approval); Pancreatic tumor	Motzer et al. (2014)
	Apatinib	VEGFR inhibitor	Approval	Gastric cancer; lung cancer; liver cancer	Roviello et al. (2016)
	MEDI9447	CD73 antibody	I	Solid tumor	Hay et al. (2016)

First, nanomaterials themselves lack active targeting and only take advantage of the EPR of tumors. However, EPR is a controversial topic in nanomedicine, and its reliability has been doubted by many researchers (Nichols and Bae, 2014). However, the accumulation of nanotherapeutics in the tumor site after intravenous injection is still limited even by the EPR effect, and most nanotherapeutics are mainly distributed in various organs (Huang et al., 2021; Dai et al., 2022). In addition, due to multiple factors, such as the high interstitial fluid pressure of the tumor and dense intercellular substances, most nanotherapeutics reaching the tumor site are blocked around the tumor and cannot penetrate into the tumor, especially vascular insufficiency at the hypoxia site (Ding et al., 2019). Therefore, nanotherapeutics should be carefully designed to have highly efficient tumor enrichment and deep penetration abilities.

Second, the biocompatibility of nanotherapeutics for alleviating tumor hypoxia in animals needs to be studied in depth. Metal-based nanoplatforms such as manganese, cerium, copper, and iron are used for oxygen delivery and generation, and non-specific accumulation in the body may be potentially toxic to normal tissues and organs (Lin et al., 2021). Although short-term (≤30 days) toxicity assessments of these nanoplatforms have been reported in most literature; however, long-term toxicity (≥6 months) still needs to be evaluated for future clinical transformation. In addition, due to the complex and variable physiological environment, nanotherapeutics may release oxygen prematurely in the circulation, which may lead to cytotoxicity to other organs. Therefore, it is important to develop nanomaterials with controlled release and monitor their biosafety. Therefore, it is of great significance to develop nanoplatforms with good biological safety and controllable oxygen release.

Third, the design of nanoscale oxygen-carrying platforms is considered suitable for clinical transformation. At present, oxygen delivery is mainly used as the source of ROS production in various oxygen-related treatments. How much oxygen can be carried by the nanoplatform, how much oxygen can be supplied for the tumor, and how long its oxygen supply can last all need to be considered. Therefore, when designing nanomaterials, it is necessary to consider the oxygen supply level, oxygen retention time and oxygen consumption required for treatment in the tumor to obtain an oxygen production/delivery nanoplatform. In addition, the design of facile synthetic nanotherapeutics is conducive to industrial production.

Fourth, nanomaterials should have imaging capabilities that can reflect hypoxia information during tumor treatment. The current polarographic Clark electrodes for measuring the partial pressure of oxygen have technical limitations, are invasive, and cannot be used for the clinical evaluation of tumor hypoxia. Immunostaining of hypoxia markers in tumor tissue only shows the hypoxic area of the tumor and does not provide accurate quantitative information in real time. The combination of imaging technologies such as CT, MRI, and positron emission computed tomography (PET) can fill the gap in tumor hypoxia assessment (Liu et al., 2017b). For example, hypoxia-sensitive fluorescent nanoprobes can be used for hypoxia monitoring. Furthermore, the combined use of these imaging modalities may provide additional valuable information about tumor physiology. Therefore, the development of nanoplatforms with hypoxia relief and imaging abilities is a future trend.

## References

[B1] Al Tameemi W. Dale T. P. Al-Jumaily R. M. K. Forsyth N. R. (2019). Hypoxia-modified cancer cell metabolism. Hypoxia-modified cancer Cell Metab. 7, 4. 10.3389/fcell.2019.00004 PMC6362613 30761299

[B2] An S. Zhang G. Li K. Huang Z. Wang X. Guo Y. (2021). Self-supporting 3D carbon nitride with tunable n→ π* electronic transition for enhanced solar hydrogen production. Adv. Mat. 33 (49), 2104361. 10.1002/adma.202104361 34632632

[B3] Belisario D. C. Kopecka J. Pasino M. Akman M. De Smaele E. Donadelli M. (2020). Hypoxia dictates metabolic rewiring of tumors: Implications for chemoresistance. Cells 9 (12), 2598. 10.3390/cells9122598 33291643 PMC7761956

[B4] Bennewith K. L. Dedhar S. (2011). Targeting hypoxic tumour cells to overcome metastasis. BMC Cancer 11, 504. 10.1186/1471-2407-11-504 22128892 PMC3247198

[B5] Briolay T. Petithomme T. Fouet M. Nguyen-Pham N. Blanquart C. Boisgerault N. (2021). Delivery of cancer therapies by synthetic and bio-inspired nanovectors. Mol. Cancer 20 (1), 55. 10.1186/s12943-021-01346-2 33761944 PMC7987750

[B6] Brown J. M. Wilson W. R. (2004). Exploiting tumour hypoxia in cancer treatment. Nat. Rev. Cancer 4 (6), 437–447. 10.1038/nrc1367 15170446

[B7] Carmeliet P. Jain R. K. (2011). Principles and mechanisms of vessel normalization for cancer and other angiogenic diseases. Nat. Rev. Drug Discov. 10 (6), 417–427. 10.1038/nrd3455 21629292

[B8] Chang J. Qin X. Li S. He F. Gai S. Ding H. (2022). Combining cobalt ferrite nanozymes with a natural enzyme to reshape the tumor microenvironment for boosted cascade enzyme-like activities. ACS Appl. Mat. Interfaces 14 (40), 45217–45228. 10.1021/acsami.2c14433 36190449

[B9] Chauhan V. P. Stylianopoulos T. Martin J. D. Popovic Z. Chen O. Kamoun W. S. (2012). Normalization of tumour blood vessels improves the delivery of nanomedicines in a size-dependent manner. Nat. Nanotechnol. 7 (6), 383–388. 10.1038/nnano.2012.45 22484912 PMC3370066

[B10] Chen D. Dai H. Wang W. Cai Y. Mou X. Zou J. (2022). Proton-driven transformable 1O2-nanotrap for dark and hypoxia tolerant photodynamic therapy. Adv. Sci. 9 (17), 2200128. 10.1002/advs.202200128 PMC9189669 35435332

[B11] Chen D. Qu X. Shao J. Wang W. Dong X. (2020). Anti-vascular nano agents: A promising approach for cancer treatment. J. Mater. Chem. B 8 (15), 2990–3004. 10.1039/c9tb02957e 32211649

[B12] Chen D. S. Hurwitz H. (2018). Combinations of bevacizumab with cancer immunotherapy. Cancer J. 24 (4), 193–204. 10.1097/PPO.0000000000000327 30119083

[B13] Chen J. Kang N. Fan J. Lu C. Lv K. (2022). Carbon nitride for photocatalytic water splitting to produce hydrogen and hydrogen peroxide. Mat. Today Chem. 26, 101028. 10.1016/j.mtchem.2022.101028

[B14] Chen J. Ma Q. Li M. Chao D. Huang L. Wu W. (2021). Glucose-oxidase like catalytic mechanism of noble metal nanozymes. Nat. Commun. 12 (1), 3375–3379. 10.1038/s41467-021-23737-1 34099730 PMC8184917

[B15] Chen M. Zhang Y. Cui L. Cao Z. Wang Y. Zhang W. (2021). Protonated 2D carbon nitride sensitized with Ce6 as a smart metal-free nanoplatform for boosted acute multimodal photo-sono tumor inactivation and long-term cancer immunotherapy. Chem. Eng. J. 422, 130089. 10.1016/j.cej.2021.130089

[B16] Chen Q. W. Qiao J. Y. Liu X. H. Zhang C. Zhang X. Z. (2021). Customized materials-assisted microorganisms in tumor therapeutics. Chem. Soc. Rev. 50 (22), 12576–12615. 10.1039/d0cs01571g 34605834

[B17] Chen T. Chu Q. Li M. Han G. Li X. (2021). Fe(3)O(4)@Pt nanoparticles to enable combinational electrodynamic/chemodynamic therapy. J. Nanobiotechnology 19 (1), 206. 10.1186/s12951-021-00957-7 34246260 PMC8272323

[B18] Chen X. Zhang X. Wu Y. Chen Y. Guo Y. Jana D. (2022). Tumor extracellular matrix-targeted nanoscavengers reverse suppressive microenvironment for cocktail therapy. Research 61. 78–90. 10.1016/j.mattod.2022.10.018

[B19] Cheng L. Wang C. Feng L. Yang K. Liu Z. (2014). Functional nanomaterials for phototherapies of cancer. Chem. Rev. 114 (21), 10869–10939. 10.1021/cr400532z 25260098

[B20] Cheng X. Sun R. Yin L. Chai Z. Shi H. Gao M. (2017). Light-triggered assembly of gold nanoparticles for photothermal therapy and photoacoustic imaging of tumors *in vivo* . Adv. Mat. 29 (6), 1604894. 10.1002/adma.201604894 27921316

[B21] Cheng Y. Cheng H. Jiang C. Qiu X. Wang K. Huan W. (2015). Perfluorocarbon nanoparticles enhance reactive oxygen levels and tumour growth inhibition in photodynamic therapy. Nat. Commun. 6, 8785. 10.1038/ncomms9785 26525216 PMC4659941

[B22] Cheng Y. Zheng R. Wu X. Xu K. Song P. Wang Y. (2021). Thylakoid membranes with unique photosystems used to simultaneously produce self-supplying oxygen and singlet oxygen for hypoxic tumor therapy. Adv. Healthc. Mater 10 (6), e2001666. 10.1002/adhm.202001666 33448152

[B23] Chu D. K. Kim L. H. Y. Young P. J. Zamiri N. Almenawer S. A. Jaeschke R. (2018). Mortality and morbidity in acutely ill adults treated with liberal versus conservative oxygen therapy (IOTA): A systematic review and meta-analysis. Lancet 391 (10131), 1693–1705. 10.1016/S0140-6736(18)30479-3 29726345

[B24] Ciaccio C. Coletta A. Coletta M. (2022). Role of hemoglobin structural-functional relationships in oxygen transport. Mol. Aspects Med. 84, 101022. 10.1016/j.mam.2021.101022 34509280

[B25] Cui X. Wen J. Hao X. Zhang S. Zhao B. (2022). HOCI probe CPP induces the differentiation of human dermal fibroblasts into vascular endothelial cells through PHD2/HIF-1α/HEY1 signaling pathway. Cells 11 (19), 3126. 10.3390/cells11193126 36231088 PMC9562224

[B26] Dai H. Cheng Z. Zhang T. Wang W. Shao J. Wang W. (2022). Boron difluoride formazanate dye for high-efficiency NIR-II fluorescence imaging-guided cancer photothermal therapy. Chin. Chem. Lett. 33 (5), 2501–2506. 10.1016/j.cclet.2021.11.079

[B27] Dai Phung C. Tran T. H. Pham L. M. Nguyen H. T. Jeong J. H. Yong C. S. (2020). Current developments in nanotechnology for improved cancer treatment, focusing on tumor hypoxia. Curr. Dev. Nanotechnol. Improv. cancer Treat. Focus. tumor hypoxia 324, 413–429. 10.1016/j.jconrel.2020.05.029 32461115

[B28] Denny W. A. (2000). The role of hypoxia-activated prodrugs in cancer therapy. Lancet Oncol. 1 (1), 25–29. 10.1016/S1470-2045(00)00006-1 11905684

[B29] Devarajan N. Manjunathan R. J.C.R.i.O.H S. K. (2021). Tumor hypoxia: The major culprit behind cisplatin resistance in cancer patients. Tumor hypoxia major culprit behind cisplatin Resist. cancer patients 162, 103327. 10.1016/j.critrevonc.2021.103327 33862250

[B30] Ding B. Zheng P. Ma P. Lin J. (2020). Manganese oxide nanomaterials: Synthesis, properties, and theranostic applications. Adv. Mater 32 (10), e1905823. 10.1002/adma.201905823 31990409

[B31] Ding J. Chen J. Gao L. Jiang Z. Zhang Y. Li M. (2019). Engineered nanomedicines with enhanced tumor penetration. Nano Today 29, 100800. 10.1016/j.nantod.2019.100800

[B32] Dong Z. Wang C. Gong Y. Zhang Y. Fan Q. Hao Y. (2022). Chemical modulation of glucose metabolism with a fluorinated CaCO3 nanoregulator can potentiate radiotherapy by programming antitumor immunity. ACS Nano 16 (9), 13884–13899. 10.1021/acsnano.2c02688 36075132

[B33] Duan X. Luo M. Li J. Shen Z. Xie K. (2022). Overcoming therapeutic resistance to platinum-based drugs by targeting Epithelial-Mesenchymal transition. Front. Oncol. 12, 1008027. 10.3389/fonc.2022.1008027 36313710 PMC9614084

[B34] Fong G. H. Takeda K. (2008). Role and regulation of prolyl hydroxylase domain proteins. Cell Death Differ. 15 (4), 635–641. 10.1038/cdd.2008.10 18259202

[B35] Fu L. H. Qi C. Lin J. Huang P. (2018). Catalytic chemistry of glucose oxidase in cancer diagnosis and treatment. Chem. Soc. Rev. 47 (17), 6454–6472. 10.1039/c7cs00891k 30024579

[B36] Fu L. H. Wan Y. Qi C. He J. Li C. Yang C. (2021). Nanocatalytic theranostics with glutathione depletion and enhanced reactive oxygen species generation for efficient cancer therapy. Adv. Mater 33 (7), e2006892. 10.1002/adma.202006892 33394515

[B37] Fu Y. Zhang X. He Y. Li X. Tang Y. (2022). Decrease in tumor interstitial pressure for enhanced drug intratumoral delivery and synergistic tumor therapy. ACS Nano 16 (11), 18376–18389. 10.1021/acsnano.2c06356 36355037

[B38] Gao F. Wu J. Gao H. Hu X. Liu L. Midgley A. C. (2020). Hypoxia-tropic nanozymes as oxygen generators for tumor-favoring theranostics. Biomaterials 230, 119635. 10.1016/j.biomaterials.2019.119635 31767443

[B39] Gao M. Liang C. Song X. Chen Q. Jin Q. Wang C. (2017). Erythrocyte-membrane-enveloped perfluorocarbon as nanoscale artificial red blood cells to relieve tumor hypoxia and enhance cancer radiotherapy. Adv. Mater 29 (35), 1701429. 10.1002/adma.201701429 28722140

[B40] Gao P. Pan W. Li N. Tang B. (2019). Boosting cancer therapy with organelle-targeted nanomaterials. ACS Appl. Mater Interfaces 11 (30), 26529–26558. 10.1021/acsami.9b01370 31136142

[B41] Gell D. A. (2018). Structure and function of haemoglobins. Blood Cells Mol. Dis. 70, 13–42. 10.1016/j.bcmd.2017.10.006 29126700

[B42] Gilkes D. M. Semenza G. L. Wirtz D. (2014). Hypoxia and the extracellular matrix: Drivers of tumour metastasis. Nat. Rev. Cancer 14 (6), 430–439. 10.1038/nrc3726 24827502 PMC4283800

[B43] Goyal M. M. Basak A. J. P. (2010). Human catalase: Looking for complete identity. Hum. catalase Look. complete identity 1 (10), 888–897. 10.1007/s13238-010-0113-z PMC4875117 21204015

[B44] Haas N. B. QuIrt I. Hotte S. McWhirtEr E. Polintan R. Litwin S. (2014). Phase II trial of vorinostat in advanced melanoma. Invest. New Drugs 32 (3), 526–534. 10.1007/s10637-014-0066-9 24464266

[B45] Hay C. M. Sult E. Huang Q. Mulgrew K. Fuhrmann S. R. McGlinchey K. A. (2016). Targeting CD73 in the tumor microenvironment with MEDI9447. Oncoimmunology 5 (8), e1208875. 10.1080/2162402X.2016.1208875 27622077 PMC5007986

[B46] He W. Zhou Y. T. Wamer W. G. Hu X. Wu X. Zheng Z. (2013). Intrinsic catalytic activity of Au nanoparticles with respect to hydrogen peroxide decomposition and superoxide scavenging. Biomaterials 34 (3), 765–773. 10.1016/j.biomaterials.2012.10.010 23103160

[B47] Hou L. Gong X. Yang J. Zhang H. Yang W. Chen X. (2022). Hybrid-membrane-decorated prussian blue for effective cancer immunotherapy via tumor-associated macrophages polarization and hypoxia relief. Adv. Mater 34 (14), e2200389. 10.1002/adma.202200389 35103352

[B48] Hu H. Qian X. Chen Y. J. S. B. (2020). Microalgae-enabled photosynthetic alleviation of tumor hypoxia for enhanced nanotherapies. Sci. Bull. 65 (22), 1869–1871. 10.1016/j.scib.2020.07.019 36738048

[B49] Huang B. Huang Y. Han H. Ge Q. Yang D. Hu Y. (2021). An NIR-II responsive nanoplatform for cancer photothermal and oxidative stress therapy. Front. Bioeng. Biotechnol. 9 (885), 751757. 10.3389/fbioe.2021.751757 34722478 PMC8553991

[B50] Huang G. Chen L. (2010). Recombinant human endostatin improves anti-tumor efficacy of paclitaxel by normalizing tumor vasculature in Lewis lung carcinoma. J. Cancer Res. Clin. Oncol. 136 (8), 1201–1211. 10.1007/s00432-010-0770-6 20130910 PMC11827984

[B51] Huang J. Zhuang C. Chen J. Chen X. Li X. Zhang T. (2022). Targeted drug/gene/photodynamic therapy via a stimuli-responsive dendritic-polymer-based nanococktail for treatment of EGFR-TKI-resistant non-small-cell lung cancer. Adv. Mater 34 (27), e2201516. 10.1002/adma.202201516 35481881

[B52] Huang W. C. Shen M. Y. Chen H. H. Lin S. C. Chiang W. H. Wu P. H. (2015). Monocytic delivery of therapeutic oxygen bubbles for dual-modality treatment of tumor hypoxia. J. Control Release 220, 738–750. 10.1016/j.jconrel.2015.09.016 26374945

[B53] Huo M. Wang L. Chen Y. Shi J. (2017). Tumor-selective catalytic nanomedicine by nanocatalyst delivery. Nat. Commun. 8 (1), 357–412. 10.1038/s41467-017-00424-8 28842577 PMC5572465

[B54] Isaakidou A. Gazouli M. Aravantinos G. Pectasides D. Theodoropoulos G. E. (2016). Prediction of response to combination chemotherapy with irinotecan in Greek patients with metastatic colorectal cancer. J. Cancer Res. Ther. 12 (1), 193–197. 10.4103/0973-1482.148654 27072236

[B55] Jagers J. Wrobeln A. Ferenz K. B. (2021). Perfluorocarbon-based oxygen carriers: From physics to physiology. Pflugers Arch. 473 (2), 139–150. 10.1007/s00424-020-02482-2 33141239 PMC7607370

[B56] Jansman M. M. T. Hosta-Rigau L. (2018). Recent and prominent examples of nano- and microarchitectures as hemoglobin-based oxygen carriers. Adv. Colloid Interface Sci. 260, 65–84. 10.1016/j.cis.2018.08.006 30177214

[B57] Jiang W. Zhang C. Ahmed A. Zhao Y. Deng Y. Ding Y. (2019). H2O2-Sensitive upconversion nanocluster bomb for tri-mode imaging-guided photodynamic therapy in deep tumor tissue. Adv. Healthc. Mat. 8 (20), 1900972. 10.1002/adhm.201900972 31566306

[B58] Jing X. Yang F. Shao C. Wei K. Xie M. Shen H. (2019). Role of hypoxia in cancer therapy by regulating the tumor microenvironment. Mol. Cancer 18 (1), 157. 10.1186/s12943-019-1089-9 31711497 PMC6844052

[B59] Kim H. Yoon J. Lee W. T. Nguyen N. T. Le X. T. (2023). Upconverting nanoparticle-containing erythrocyte-sized hemoglobin microgels that generate heat, oxygen and reactive oxygen species for suppressing hypoxic tumors. Bioact. Mater. 22, 112–126. 10.1016/j.bioactmat.2022.09.020 36203958 PMC9526021

[B60] Komkova M. A. Karyakin A. A. (2022). Prussian blue: From advanced electrocatalyst to nanozymes defeating natural enzyme. Mikrochim. Acta 189 (8), 290. 10.1007/s00604-022-05363-w 35879483

[B61] Koo S. Park O. K. Kim J. Han S. I. Yoo T. Y. Lee N. (2022). Enhanced chemodynamic therapy by Cu–Fe peroxide nanoparticles: Tumor microenvironment-mediated synergistic Fenton reaction. ACS Nano 16 (2), 2535–2545. 10.1021/acsnano.1c09171 35080370

[B62] Kopecka J. Porto S. Lusa S. Gazzano E. Salzano G. Giordano A. (2015). Self-assembling nanoparticles encapsulating zoledronic acid revert multidrug resistance in cancer cells. Oncotarget 6 (31), 31461–31478. 10.18632/oncotarget.5058 26372812 PMC4741618

[B63] Kopecka J. Salaroglio I. C. Perez-Ruiz E. Sarmento-Ribeiro A. B. Saponara S. Riganti C. (2021). Hypoxia as a driver of resistance to immunotherapy. Drug Resist Updat 59, 100787. 10.1016/j.drup.2021.100787 34840068

[B64] Li H. Liu Y. Huang B. Zhang C. Wang Z. She W. (2022). Highly efficient GSH-responsive "Off-On" NIR-II fluorescent Fenton nanocatalyst for multimodal imaging-guided photothermal/chemodynamic synergistic cancer therapy. Anal. Chem. 94 (29), 10470–10478. 10.1021/acs.analchem.2c01738 35816734

[B65] Li K. Gong Y. Qiu D. Tang H. Zhang J. Yuan Z. (2022). Hyperbaric oxygen facilitates teniposide-induced cGAS-STING activation to enhance the antitumor efficacy of PD-1 antibody in HCC. J. Immunother. Cancer 10 (8), e004006. 10.1136/jitc-2021-004006 36002188 PMC9413187

[B66] Li L. Yang Z. Fan W. He L. Cui C. Zou J. (2020). *In situ* polymerized hollow mesoporous organosilica biocatalysis nanoreactor for enhancing ROS-mediated anticancer therapy. Adv. Funct. Mat. 30 (4), 1907716. 10.1002/adfm.201907716 PMC7546450 33041745

[B67] Li Q. Hang L. Jiang W. Dou J. Xiao L. Tang X. (2020). Pre- and post-irradiation mild hyperthermia enabled by NIR-II for sensitizing radiotherapy. Biomaterials 257, 120235. 10.1016/j.biomaterials.2020.120235 32736260

[B68] Li S. Lui K. H. Lau W. S. Chen J. Lo W. S. Li X. (2022). MSOT-guided nanotheranostics for synergistic mild photothermal therapy and chemotherapy to boost necroptosis/apoptosis. ACS Appl. Mater Interfaces 14, 33712–33725. 10.1021/acsami.2c07592 35822699

[B69] Li T. Mao C. Wang X. Shi Y. Tao Y. (2020). Epigenetic crosstalk between hypoxia and tumor driven by HIF regulation. J. Exp. Clin. Cancer Res. 39 (1), 224–225. 10.1186/s13046-020-01733-5 33109235 PMC7592369

[B70] Li W. Zhao X. Du B. Li X. Liu S. Yang X. Y. (2016). Gold nanoparticle-mediated targeted delivery of recombinant human endostatin normalizes tumour vasculature and improves cancer therapy. Sci. Rep. 6, 30619. 10.1038/srep30619 27470938 PMC4965746

[B71] Li W. Zhong D. Hua S. Du Z. Zhou M. (2020). Biomineralized biohybrid algae for tumor hypoxia modulation and cascade radio-photodynamic therapy. ACS Appl. Mater Interfaces 12 (40), 44541–44553. 10.1021/acsami.0c14400 32935973

[B72] Li X. F. Carlin S. Urano M. Russell J. Ling C. C. O'Donoghue J. A. (2007). Visualization of hypoxia in microscopic tumors by immunofluorescent microscopy. Cancer Res. 67 (16), 7646–7653. 10.1158/0008-5472.CAN-06-4353 17699769

[B73] Li X. Kwon N. Guo T. Liu Z. Yoon J. (2018). Innovative strategies for hypoxic-tumor photodynamic therapy. Angew. Chem. Int. Ed. Engl. 57 (36), 11522–11531. 10.1002/anie.201805138 29808948

[B74] Liang M. Yan X. J. A. O. C. R. Nanozymes (2019). Nanozymes: From new concepts, mechanisms, and standards to applications. Acc. Chem. Res. 52 (8), 2190–2200. 10.1021/acs.accounts.9b00140 31276379

[B75] Liang X. Chen M. Bhattarai P. Hameed S. Dai Z. (2020). Perfluorocarbon@Porphyrin nanoparticles for tumor hypoxia relief to enhance photodynamic therapy against liver metastasis of colon cancer. ACS Nano 14 (10), 13569–13583. 10.1021/acsnano.0c05617 32915537

[B76] Liao G. He F. Li Q. Zhong L. Zhao R. Che H. (2020). Emerging graphitic carbon nitride-based materials for biomedical applications. Prog. Mater. Sci. 112, 100666. 10.1016/j.pmatsci.2020.100666

[B77] Lin X. Hu Y. l. Zhang C. Yin J. Cui R. Yang D. l. (2021). More severe toxicity of gold nanoparticles with rougher surface in mouse hippocampal neurons. J. Central South Univ. 28 (12), 3642–3653. 10.1007/s11771-021-4844-1

[B78] Lin Y. Ren J. Qu X. J. A. m. (2014). Nano-gold as artificial enzymes: Hidden talents. Adv. Mat. 26 (25), 4200–4217. 10.1002/adma.201400238 24692212

[B79] Liu B. Bian Y. Yuan M. Zhu Y. Liu S. Ding H. (2022). L-buthionine sulfoximine encapsulated hollow calcium peroxide as a chloroperoxidase nanocarrier for enhanced enzyme dynamic therapy. Biomaterials 289, 121746. 10.1016/j.biomaterials.2022.121746 36084482

[B80] Liu C. P. Wu T. H. Chen K. C. Chen Y. X. Chen G. S. (2017). Self-supplying O2 through the catalase-like activity of gold nanoclusters for photodynamic therapy against hypoxic cancer cells. Small 13 (26), 1700278. 10.1002/smll.201700278 28509427

[B81] Liu H. Lv X. Qian J. Li H. Qian Y. Wang X. (2020). Graphitic carbon nitride quantum dots embedded in carbon nanosheets for near-infrared imaging-guided combined photo-chemotherapy. ACS Nano 14 (10), 13304–13315. 10.1021/acsnano.0c05143 33016066

[B82] Liu J.-n. Bu W. Shi J. J. C. r. (2017). Chemical design and synthesis of functionalized probes for imaging and treating tumor hypoxia. Chem. Rev. 117 (9), 6160–6224. 10.1021/acs.chemrev.6b00525 28426202

[B83] Liu P. Xie X. Shi X. Peng Y. Ding J. Zhou W. (2019). Oxygen-self-Supplying and HIF-1α-Inhibiting core-shell nanosystem for hypoxia-resistant photodynamic therapy. ACS Appl. Mater Interfaces 11 (51), 48261–48270. 10.1021/acsami.9b18112 31763809

[B84] Liu P. Zhang H. Wu X. Guo L. Wang F. Xia G. (2016). Tf-PEG-PLL-PLGA nanoparticles enhanced chemosensitivity for hypoxia-responsive tumor cells. Onco Targets Ther. 9, 5049–5059. 10.2147/OTT.S108169 27574446 PMC4990384

[B85] Liu X. Pan Y. Yang J. Gao Y. Huang T. Luan X. (2020). Gold nanoparticles doped metal-organic frameworks as near-infrared light-enhanced cascade nanozyme against hypoxic tumors. Nano Res. 13 (3), 653–660. 10.1007/s12274-020-2668-1

[B86] Liu X. Ye N. Liu S. Guan J. Deng Q. Zhang Z. (2021). Hyperbaric oxygen boosts PD-1 antibody delivery and T cell infiltration for augmented immune responses against solid tumors. Adv. Sci. (Weinh) 8 (15), e2100233. 10.1002/advs.202100233 34085419 PMC8336507

[B87] Liu Y. Guo Q. Zhu X. Feng W. Wang L. (2016). Optimization of prussian blue coated NaDyF_4_: X% Lu nanocomposites for multifunctional imaging-guided photothermal therapy. Full Pap. 26 (28), 5120–5130. 10.1002/adfm.201601478

[B88] Lou-Franco J. Das B. Elliott C. Cao C. (2021). Gold nanozymes: From concept to biomedical applications. Nanomicro. Lett. 13 (1), 10–36. 10.1007/s40820-020-00532-z PMC8187695 34138170

[B89] Lu N. Fan W. Yi X. Wang S. Wang Z. Tian R. (2018). Biodegradable hollow mesoporous organosilica nanotheranostics for mild hyperthermia-induced bubble-enhanced oxygen-sensitized radiotherapy. ACS Nano 12 (2), 1580–1591. 10.1021/acsnano.7b08103 29384652

[B90] Lu Z. Gao J. Fang C. Zhou Y. Li X. Han G. (2020). Porous Pt nanospheres incorporated with GOx to enable synergistic oxygen-inductive starvation/electrodynamic tumor therapy. Adv. Sci. (Weinh) 7 (17), 2001223. 10.1002/advs.202001223 32995127 PMC7507307

[B91] Luo Z. Zhou M. Wang X. J. A. C. B. E. (2018). Cobalt-based cubane molecular co-catalysts for photocatalytic water oxidation by polymeric carbon nitrides. Appl. Catal. B Environ. 238, 664–671. 10.1016/j.apcatb.2018.07.056

[B92] Ma J. Peng X. Zhou Z. Yang H. Wu K. Fang Z. (2022). Extended conjugation tuning carbon nitride for non-sacrificial H2O2 photosynthesis and hypoxic tumor therapy. Angew. Chem. Int. Ed. Engl. 61 (43), e202210856. 10.1002/anie.202210856 35939064

[B93] Ma Y. Zhao Y. Bejjanki N. K. Tang X. Jiang W. Dou J. (2019). Nanoclustered cascaded enzymes for targeted tumor starvation and deoxygenation-activated chemotherapy without systemic toxicity. Nanoclustered cascaded Enzym. Target. tumor starvation deoxygenation-activated Chemother. without Syst. Toxic. 13 (8), 8890–8902. 10.1021/acsnano.9b02466 31291092

[B94] Mackenzie C. F. Dube G. P. Pitman A. Zafirelis M. (2019). Users guide to pitfalls and lessons learned about HBOC-201 during clinical trials, expanded access, and clinical use in 1,701 patients. Shock 52 (1), 92–99. 10.1097/SHK.0000000000001038 29076972

[B95] Malaviya P. Shukal D. Vasavada A. R. (2019). Nanotechnology-based drug delivery, metabolism and toxicity. Curr. Drug Metab. 20 (14), 1167–1190. 10.2174/1389200221666200103091753 31902350

[B96] Matei D. Schilder J. Sutton G. Perkins S. Breen T. Quon C. (2009). Activity of 2 methoxyestradiol (panzem NCD) in advanced, platinum-resistant ovarian cancer and primary peritoneal carcinomatosis: A hoosier oncology group trial. Gynecol. Oncol. 115 (1), 90–96. 10.1016/j.ygyno.2009.05.042 19577796

[B97] Matuszewska K. Pereira M. Petrik D. Lawler J. Petrik J. (2021). Normalizing tumor vasculature to reduce hypoxia, enhance perfusion, and optimize therapy uptake. Cancers 13 (17), 4444. 10.3390/cancers13174444 34503254 PMC8431369

[B98] McAleese C. E. Choudhury C. Butcher N. J. Minchin R. F. (2021). Hypoxia-mediated drug resistance in breast cancers. Cancer Lett. 502, 189–199. 10.1016/j.canlet.2020.11.045 33278499

[B99] Motzer R. J. Barrios C. H. Kim T. M. Falcon S. Cosgriff T. Harker W. G. (2014). Phase II randomized trial comparing sequential first-line everolimus and second-line sunitinib versus first-line sunitinib and second-line everolimus in patients with metastatic renal cell carcinoma. J. Clin. Oncol. 32 (25), 2765–2772. 10.1200/JCO.2013.54.6911 25049330 PMC5569681

[B100] Mu J. Du Y. Li X. Yan R. Zhong H. Cai M. (2023). Collagen-anchored cascade nanoreactors with prolonged intratumoral retention for combined cancer starvation and chemotherapy. Chem. Eng. J. 451, 138554. 10.1016/j.cej.2022.138554

[B101] Murdoch C. Giannoudis A. Lewis C. E. (2004). Mechanisms regulating the recruitment of macrophages into hypoxic areas of tumors and other ischemic tissues. Blood 104 (8), 2224–2234. 10.1182/blood-2004-03-1109 15231578

[B102] Nichols J. W. Bae Y. H. (2014). Epr: Evidence and fallacy. J. Control Release 190, 451–464. 10.1016/j.jconrel.2014.03.057 24794900

[B103] O'Donnell J. L. Joyce M. R. Shannon A. M. Harmey J. Geraghty J. Bouchier-Hayes D. (2006). Oncological implications of hypoxia inducible factor-1alpha (HIF-1alpha) expression. Cancer Treat. Rev. 32 (6), 407–416. 10.1016/j.ctrv.2006.05.003 16889900

[B104] Oki Y. Copeland A. Romaguera J. Fayad L. Fanale M. Faria S. d. C. (2012). Clinical experience with the heat shock protein-90 inhibitor, tanespimycin, in patients with relapsed lymphoma. Leuk. Lymphoma 53 (5), 990–992. 10.3109/10428194.2011.631236 21988665

[B105] Olson J. S. (2022). Kinetic mechanisms for O2 binding to myoglobins and hemoglobins. Mol. Asp. Med. 84, 101024. 10.1016/j.mam.2021.101024 PMC8821315 34544605

[B106] Pan Y. Zhu Y. Xu C. Pan C. Shi Y. Zou J. (2022). Biomimetic yolk-shell nanocatalysts for activatable dual-modal-image-guided triple-augmented chemodynamic therapy of cancer. ACS Nano 16 (11), 19038–19052. 10.1021/acsnano.2c08077 36315056

[B107] Petrova V. Annicchiarico-Petruzzelli M. Melino G. Amelio I. (2018). The hypoxic tumour microenvironment. Rev. Artic. 7 (1), 1–13. 10.1038/s41389-017-0011-9 PMC5833859 29362402

[B108] Poluzzi C. Iozzo R. V. Schaefer L. (2016). Endostatin and endorepellin: A common route of action for similar angiostatic cancer avengers. Adv. Drug Deliv. Rev. 97, 156–173. 10.1016/j.addr.2015.10.012 26518982 PMC4753091

[B109] Qiao Y. Yang F. Xie T. Du Z. Zhong D. Qi Y. (2020). Engineered algae: A novel oxygen-generating system for effective treatment of hypoxic cancer. Sci. Adv. 6 (21), eaba5996. 10.1126/sciadv.aba5996 32490207 PMC7239646

[B110] Qin X. Wu C. Niu D. Qin L. Wang X. Wang Q. (2021). Peroxisome inspired hybrid enzyme nanogels for chemodynamic and photodynamic therapy. Nat. Commun. 12 (1), 5243–5315. 10.1038/s41467-021-25561-z 34475406 PMC8413279

[B111] Rankin E. B. Giaccia A. J. (2016). Hypoxic control of metastasis. Science 352 (6282), 175–180. 10.1126/science.aaf4405 27124451 PMC4898055

[B112] Rankin E. B. Giaccia A. J. J. S. (2016). Hypoxic control of metastasis. Hypoxic control metastasis 352 (6282), 175–180. 10.1126/science.aaf4405 PMC4898055 27124451

[B113] Riganti C. Castella B. Kopecka J. Campia I. Coscia M. Pescarmona G. (2013). Zoledronic acid restores doxorubicin chemosensitivity and immunogenic cell death in multidrug-resistant human cancer cells. PLoS One 8 (4), e60975. 10.1371/journal.pone.0060975 23593363 PMC3625183

[B114] Rose C. Lustig R. McINtosh N. Teicher B. (1986). A clinical trial of Fluosol DA 20% in advanced squamous cell carcinoma of the head and neck. Int. J. Radiat. Oncol. Biol. Phys. 12 (8), 1325–1327. 10.1016/0360-3016(86)90164-1 3759553

[B115] Roviello G. Ravelli A. Fiaschi A. I. Cappelletti M. R. Gobbi A. Senti C. (2016). Apatinib for the treatment of gastric cancer. Expert Rev. Gastroenterol. Hepatol. 10 (8), 887–892. 10.1080/17474124.2016.1209407 27376400

[B116] Rubinstein M. M. Goss C. Avecilla S. T. Dube G. P. Riely G. J. Mones J. V. (2020). Management of thymoma-associated pure red cell aplasia: A novel use of blood substitute HBOC-201 in a jehovah's witness. Clin. Case Rep. 8 (2), 289–292. 10.1002/ccr3.2626 32128175 PMC7044386

[B117] Schwarte L. A. Schober P. Loer S. A. J. C. O. I. A. (2019). Benefits and harms of increased inspiratory oxygen concentrations. Curr. Opin. Anaesthesiol. 32 (6), 783–791. 10.1097/ACO.0000000000000791 31464698

[B118] Singleton D. C. Macann A. Wilson W. R. (2021). Therapeutic targeting of the hypoxic tumour microenvironment. Nat. Rev. Clin. Oncol. 18 (12), 751–772. 10.1038/s41571-021-00539-4 34326502

[B119] Solit D. B. Osman I. Polsky D. Panageas K. S. Daud A. Goydos J. S. (2008). Phase II trial of 17-allylamino-17-demethoxygeldanamycin in patients with metastatic melanoma. Clin. Cancer Res. 14 (24), 8302–8307. 10.1158/1078-0432.CCR-08-1002 19088048 PMC2629404

[B120] Song G. Liang C. Gong H. Zheng X. Cheng L. (2015). Core-shell MnSe@Bi2 Se3 fabricated via a cation exchange method as novel nanotheranostics for multimodal imaging and synergistic thermoradiotherapy. Adv. Mater 27 (40), 6110–6117. 10.1002/adma.201503006 26331476

[B121] Song X. Feng L. Liang C. Yang K. Liu Z. (2016). Ultrasound triggered tumor oxygenation with oxygen-shuttle nanoperfluorocarbon to overcome hypoxia-associated resistance in cancer therapies. Nano Lett. 16 (10), 6145–6153. 10.1021/acs.nanolett.6b02365 27622835

[B122] Telarovic I. Wenger R. H. Pruschy M. (2021). Interfering with tumor hypoxia for radiotherapy optimization. J. Exp. Clin. Cancer Res. 40 (1), 197–226. 10.1186/s13046-021-02000-x 34154610 PMC8215813

[B123] Teng Z. Zhang Q. Yang H. Kato K. Yang W. Lu Y. R. (2021). Atomically dispersed antimony on carbon nitride for the artificial photosynthesis of hydrogen peroxide. Nat. Catal. 4 (5), 374–384. 10.1038/s41929-021-00605-1

[B124] Tian B. Wang C. Du Y. Dong S. Feng L. Liu B. (2022). Near infrared-triggered theranostic nanoplatform with controlled release of HSP90 inhibitor for synergistic mild photothermal and enhanced nanocatalytic therapy with hypoxia relief. Small 18 (28), e2200786. 10.1002/smll.202200786 35661402

[B125] Tian H. Luo Z. Liu L. Zheng M. Chen Z. (2017). Cancer cell membrane-biomimetic oxygen nanocarrier for breaking hypoxia-induced chemoresistance. Adv. Funct. Mater. 27 (38), 1703197. 10.1002/adfm.201703197

[B126] Unger E. C. Lickliter J. D. Ruben J. Jennens R. Kichenadasse G. Gzell C. (2017). A phase Ib/II clinical trial of a novel oxygen therapeutic in chemoradiation of glioblastoma. J. Clin. Oncol. 35 (15), 2561. 10.1200/jco.2017.35.15_suppl.2561

[B127] Vandooren J. Opdenakker G. Loadman P. M. Edwards D. R. (2016). Proteases in cancer drug delivery. Adv. Drug Deliv. Rev. 97, 144–155. 10.1016/j.addr.2015.12.020 26756735

[B128] Vaupel P. Multhoff G. (2018). Hypoxia-/HIF-1α-Driven factors of the tumor microenvironment impeding antitumor immune responses and promoting malignant progression. Adv. Exp. Med. Biol. 1072, 171–175. 10.1007/978-3-319-91287-5_27 30178341

[B129] Vaupel P. Schlenger K. Knoop C. Hockel M. (1991). Oxygenation of human tumors: Evaluation of tissue oxygen distribution in breast cancers by computerized O2 tension measurements. Cancer Res. 51 (12), 3316–3322. 2040005

[B130] Velcheva V. Hegetschweiler K. Momekov G. Ivanova S. Ugrinov A. Morgenstern B. (2022). Platinum(IV) complexes of the 1,3,5-triamino analogue of the biomolecule cis-inositol designed as innovative antineoplastic drug candidates. Pharmaceutics 14 (10), 2057. 10.3390/pharmaceutics14102057 36297500 PMC9611922

[B131] Wang H. Guo Y. Wang C. Jiang X. Liu H. Yuan A. (2021). Light-controlled oxygen production and collection for sustainable photodynamic therapy in tumor hypoxia. Biomaterials 269, 120621. 10.1016/j.biomaterials.2020.120621 33383301

[B132] Wang J. Soto F. Liu S. Yin Q. Purcell E. Zeng Y. (2022). Volbots: Volvox microalgae-based robots for multimode precision imaging and therapy. Res. Article 32 (50), 2201800. 10.1002/adfm.202201800.

[B133] Wang J. Ye J. Lv W. Liu S. Zhang Z. Xu J. (2022). Biomimetic nanoarchitectonics of hollow mesoporous copper oxide-based nanozymes with cascade catalytic reaction for near infrared-II reinforced photothermal-catalytic therapy. ACS Appl. Mater Interfaces 14 (36), 40645–40658. 10.1021/acsami.2c11634 36040363

[B134] Wang M. Chang M. Li C. Chen Q. Hou Z. Xing B. (2022). Tumor-microenvironment-activated reactive oxygen species amplifier for enzymatic cascade cancer starvation/chemodynamic/immunotherapy. Adv. Mat. 34 (4), 2106010. 10.1002/adma.202106010 34699627

[B135] Wang M. Zhang W. Ding B. Ma P. Lin J. (2022). Copper peroxides based multiple tumor microenvironment regulation for enhanced photodynamic/chemodynamic synergistic therapy. Res. Article 2202040. 10.1002/adom.202202040

[B136] Wang S. Wang Z. Li Z. Zhang X. Zhang H. Zhang T. (2022). Amelioration of systemic antitumor immune responses in cocktail therapy by immunomodulatory nanozymes. Sci. Adv. 8 (21), eabn3883. 10.1126/sciadv.abn3883 35622914 PMC9140981

[B137] Wang W. Cheng Y. Yu P. Wang H. Zhang Y. Xu H. (2019). Perfluorocarbon regulates the intratumoural environment to enhance hypoxia-based agent efficacy. Nat. Commun. 10 (1), 1580. 10.1038/s41467-019-09389-2 30952842 PMC6450981

[B138] Wang W. Zheng H. Jiang J. Li Z. Jiang D. Shi X. (2022). Engineering micro oxygen factories to slow tumour progression via hyperoxic microenvironments. Nat. Commun. 13 (1), 4495. 10.1038/s41467-022-32066-w 35918337 PMC9345862

[B139] Wang X. Cheng L. J. C. C. R. (2020). Multifunctional Prussian blue-based nanomaterials: Preparation, modification, and theranostic applications. Review 419, 213393. 10.1016/j.ccr.2020.213393

[B140] Wang X. Zhong X. Liu Z. Cheng L. (2020). Recent progress of chemodynamic therapy-induced combination cancer therapy. Nano Today 35, 100946. 10.1016/j.nantod.2020.100946

[B141] Wang Y. Yu J. Luo Z. Shi Q. Liu G. Wu F. (2021). Engineering endogenous tumor-associated macrophage-targeted biomimetic nano-RBC to reprogram tumor immunosuppressive microenvironment for enhanced chemo-immunotherapy. Adv. Mater 33 (39), e2103497. 10.1002/adma.202103497 34387375

[B142] Wang Z. Sun Z. Wang S. Zhu S. (2020). Visualization nanozyme based on tumor microenvironment “unlocking” for intensive combination therapy of breast cancer . Sci. Adv. 6 (48), eabc8733. 10.1126/sciadv.abc8733 33246959 PMC7695480

[B143] Wei C. Liu Y. Zhu X. Chen X. Zhou Y. Yuan G. (2020). Iridium/ruthenium nanozyme reactors with cascade catalytic ability for synergistic oxidation therapy and starvation therapy in the treatment of breast cancer. Biomaterials 238, 119848. 10.1016/j.biomaterials.2020.119848 32062149

[B144] Wei F. Kuang S. Rees T. W. Liao X. Liu J. Luo D. (2021). Ruthenium (II) complexes coordinated to graphitic carbon nitride: Oxygen self-sufficient photosensitizers which produce multiple ROS for photodynamic therapy in hypoxia. Biomaterials 276, 121064. 10.1016/j.biomaterials.2021.121064 34391019

[B145] Wei H. Wang E. J. C. S. R. (2013). Nanomaterials with enzyme-like characteristics (nanozymes): Next-generation artificial enzymes. Chem. Soc. Rev. 42 (14), 6060–6093. 10.1039/c3cs35486e 23740388

[B146] Wu T. Liu Y. Cao Y. Liu Z. (2022). Engineering macrophage exosome disguised biodegradable nanoplatform for enhanced sonodynamic therapy of glioblastoma. Adv. Mat. 34 (15), 2110364. 10.1002/adma.202110364 35133042

[B147] Wu X. Zhu Y. Huang W. Li J. Zhang B. Li Z. (2018). Hyperbaric oxygen potentiates doxil antitumor efficacy by promoting tumor penetration and sensitizing cancer cells. Adv. Sci. 5 (8), 1700859. 10.1002/advs.201700859 PMC6097095 30128223

[B148] Xiao T. Xu F. Fan Y. Jia B. Shen M. (2021). Macrophage membrane-camouflaged responsive polymer nanogels enable magnetic resonance imaging-guided chemotherapy/chemodynamic therapy of orthotopic glioma. ACS Nano 15 (12), 20377–20390. 10.1021/acsnano.1c08689 34860014

[B149] Xu G. Du X. Wang W. Qu Y. Liu X. Zhao M. (2022). Plasmonic nanozymes: Leveraging localized surface plasmon resonance to boost the enzyme-mimicking activity of nanomaterials. Review 18 (49), 2204131. 10.1002/smll.202204131. 36161698

[B150] Xu L. Wang J. Lu S. Y. Wang X. Cao Y. Wang M. (2019). Construction of a polypyrrole-based multifunctional nanocomposite for dual-modal imaging and enhanced synergistic phototherapy against cancer cells. Langmuir. 35 (28), 9246–9254. 10.1021/acs.langmuir.9b01387 31251628

[B151] Xu Q. Zhang Y. Yang Z. Jiang G. Lv M. Wang H. (2022). Tumor microenvironment-activated single-atom platinum nanozyme with H(2)O(2) self-supplement and O(2)-evolving for tumor-specific cascade catalysis chemodynamic and chemoradiotherapy. Theranostics 12 (11), 5155–5171. 10.7150/thno.73039 35836808 PMC9274735

[B152] Xu X.-X. Chen S. Y. Yi N. B. Li X. Chen S. L. Lei Z. (2022). Research progress on tumor hypoxia-associative nanomedicine. J. Control. Release 350, 829–840. 10.1016/j.jconrel.2022.09.003 36100192

[B153] Xu Y. Liu S. Y. Zeng L. Ma H. Zhang Y. Yang H. (2022). An enzyme-engineered nonporous copper(I) coordination polymer nanoplatform for cuproptosis-based synergistic cancer therapy. Adv. Mater 34 (43), e2204733. 10.1002/adma.202204733 36054475

[B154] Yan J. Shan C. Liang C. Han J. He B. Sun Y. (2022). Smart multistage “trojan horse”-inspired bovine serum albumin-coated Liposomes for enhancing tumor Penetration and antitumor efficacy . Biomacromolecules 23, 5202–5212. 10.1021/acs.biomac.2c00984 36287618

[B155] Yan J. Zhang Z. Zhan X. Chen K. Pu Y. Liang Y. (2021). *In situ* injection of dual-delivery PEG based MMP-2 sensitive hydrogels for enhanced tumor penetration and chemo-immune combination therapy. Nanoscale 13 (21), 9577–9589. 10.1039/d1nr01155c 33998643

[B156] Yang G. Tian J. Chen C. Jiang D. Xue Y. Wang C. (2019). An oxygen self-sufficient NIR-responsive nanosystem for enhanced PDT and chemotherapy against hypoxic tumors. Chem. Sci. 10 (22), 5766–5772. 10.1039/c9sc00985j 31293763 PMC6568044

[B157] Yang G. Xu L. Chao Y. Xu J. Sun X. Wu Y. (2017). Hollow MnO(2) as a tumor-microenvironment-responsive biodegradable nano-platform for combination therapy favoring antitumor immune responses. Nat. Commun. 8 (1), 902. 10.1038/s41467-017-01050-0 29026068 PMC5638920

[B158] Yang J. Li W. Luo L. Jiang M. Zhu C. Qin B. (2018). Hypoxic tumor therapy by hemoglobin-mediated drug delivery and reversal of hypoxia-induced chemoresistance. Biomaterials 182, 145–156. 10.1016/j.biomaterials.2018.08.004 30121013

[B159] Yang M. Cong C. Bian J. Xu Z. Liu X. Liu L. (2021). Photothermal controlled oxygen self-supplying “nano-bombs” via lysosome burst for transcytosis delivery and anti-tumor therapy . Appl. Mater. Today 22, 100940. 10.1016/j.apmt.2021.100940

[B160] Ye P. Li F. Zou J. Luo Y. Wang S. Lu G. (2022). *In situ* generation of gold nanoparticles on bacteria-derived magnetosomes for imaging-guided starving/chemodynamic/photothermal synergistic therapy against cancer. Situ Generation Gold Nanoparticles Bacteria-Derived Magnetosomes Imaging-Guided Starving/Chemodynamic/Photothermal Synerg. Ther. against Cancer 32 (17), 2110063. 10.1002/adfm.202110063

[B161] You Q. Zhang K. Liu J. Liu C. Wang H. Wang M. (2020). Persistent regulation of tumor hypoxia microenvironment via a bioinspired Pt-based oxygen nanogenerator for multimodal imaging-guided synergistic phototherapy. Adv. Sci. (Weinh) 7 (17), 1903341. 10.1002/advs.201903341 32995114 PMC7507529

[B162] You Y. Zhao Z. He L. Sun Z. Zhang D. Shi C. (2020). Long-term oxygen storage nanosystem for near-infrared light-triggered oxygen supplies to antagonize hypoxia-induced therapeutic resistance in nasopharyngeal carcinoma. Adv. Funct. Mater. 30 (27), 2002369. 10.1002/adfm.202002369

[B163] Yu H. Cheng Y. Wen C. Sun Y. Q. Yin X. B. (2022). Triple cascade nanocatalyst with laser-activatable O2 supply and photothermal enhancement for effective catalytic therapy against hypoxic tumor. Biomaterials 280, 121308. 10.1016/j.biomaterials.2021.121308 34896860

[B164] Yuan C. S. Deng Z. W. Qin D. Mu Y. Z. Chen X. G. Liu Y. (2021). Hypoxia-modulatory nanomaterials to relieve tumor hypoxic microenvironment and enhance immunotherapy: Where do we stand? Acta Biomater. 125, 1–28. 10.1016/j.actbio.2021.02.030 33639310

[B165] Yuan M. Liang S. Zhou Y. Xiao X. Liu B. Yang C. (2021). A robust oxygen-carrying hemoglobin-based natural sonosensitizer for sonodynamic cancer therapy. Nano Lett. 21 (14), 6042–6050. 10.1021/acs.nanolett.1c01220 34254814

[B166] Zai W. Kang L. Dong T. Wang H. Yin L. Gan S. (2021). *E. coli* membrane vesicles as a catalase carrier for long-term tumor hypoxia relief to enhance radiotherapy. ACS Nano 15 (9), 15381–15394. 10.1021/acsnano.1c07621 34520168

[B167] Zhang J. Su X. Weng L. Tang K. Miao Y. Teng Z. (2022). Gadolinium-hybridized mesoporous organosilica nanoparticles with high magnetic resonance imaging performance for targeted drug delivery. J. Colloid Interface Sci. 633, 102–112. 10.1016/j.jcis.2022.11.085 36436344

[B168] Zhang M. S. Cui J. D. Lee D. Yuen V. W. H. Chiu D. K. C. Goh C. C. (2022). Hypoxia-induced macropinocytosis represents a metabolic route for liver cancer. Nat. Commun. 13 (1), 954. 10.1038/s41467-022-28618-9 35177645 PMC8854584

[B169] Zhang M. Shen B. Song R. Wang H. Lv B. Meng X. (2019). Radiation-assisted metal ion interference tumor therapy by barium peroxide-based nanoparticles. Mat. Horiz. 6 (5), 1034–1040. 10.1039/c8mh01554f

[B170] Zhang S. Li Z. Wang Q. Liu Q. Yuan W. Feng W. (2022). An NIR-II photothermally triggered "oxygen bomb" for hypoxic tumor programmed cascade therapy. Adv. Mater 34 (29), e2201978. 10.1002/adma.202201978 35606680

[B171] Zhang X. He C. Xiang G. (2022). Engineering nanomedicines to inhibit hypoxia-inducible Factor-1 for cancer therapy. Cancer Lett. 530, 110–127. 10.1016/j.canlet.2022.01.012 35041892

[B172] Zhang Y. Kang S. Lin H. Chen M. Li Y. Cui L. (2022). Regulation of zeolite-derived upconversion photocatalytic system for near infrared light/ultrasound dual-triggered multimodal melanoma therapy under a boosted hypoxia relief tumor microenvironment via autophagy. Chem. Eng. J. 429, 132484. 10.1016/j.cej.2021.132484

[B173] Zhang Z. Wang Z. Xiong Y. Wang C. Deng Q. Yang T. (2022). A two-pronged strategy to alleviate tumor hypoxia and potentiate photodynamic therapy by mild hyperthermia. Biomater. Sci. 11 (1), 108–118. 10.1039/d2bm01691e 36468355

[B174] Zhao J. Du F. Luo Y. Shen G. Zheng F. Xu B. (2015). The emerging role of hypoxia-inducible factor-2 involved in chemo/radioresistance in solid tumors. Cancer Treat. Rev. 41 (7), 623–633. 10.1016/j.ctrv.2015.05.004 25981453

[B175] Zheng D. Li B. Xu L. Zhang Q. L. Fan J. X. Li C. X. (2018). Normalizing tumor microenvironment based on photosynthetic abiotic/biotic nanoparticles. ACS Nano 12 (6), 6218–6227. 10.1021/acsnano.8b02977 29791792

[B176] Zhong D. Li W. Hua S. Qi Y. Xie T. Qiao Y. (2021). Calcium phosphate engineered photosynthetic microalgae to combat hypoxic-tumor by *in-situ* modulating hypoxia and cascade radio-phototherapy. Theranostics 11 (8), 3580–3594. 10.7150/thno.55441 33664849 PMC7914342

[B177] Zhong D. Li W. Qi Y. He J. Zhou M. (2020). Photosynthetic biohybrid nanoswimmers system to alleviate tumor hypoxia for FL/PA/MR imaging-guided enhanced radio-photodynamic synergetic therapy. Adv. Funct. Mat. 30 (17), 1910395. 10.1002/adfm.201910395

[B178] Zhou G. Li M. J. A. M. (2022). Near-infrared-II plasmonic trienzyme-integrated metal–organic frameworks with high-efficiency enzyme cascades for synergistic trimodal oncotherapy. Adv. Mater 34, e2200871. 10.1002/adma.202200871 35429080

[B179] Zhou H. Li X. Niu D. Li Y. Liu X. (2021). Ultrasensitive chemodynamic therapy: Bimetallic peroxide triggers high pH-activated, synergistic effect/H _2_ O _2_ self-supply-mediated cascade Fenton chemistry. Adv. Healthc. Mater 10 (9), 2002126. 10.1002/adhm.202002126 33644985

[B180] Zhou J. Y. Wang W. J. Zhang C. Y. Ling Y. Y. Hong X. J. Su Q. (2022). Ru(II)-modified TiO(2) nanoparticles for hypoxia-adaptive photo-immunotherapy of oral squamous cell carcinoma. Biomaterials 289, 121757. 10.1016/j.biomaterials.2022.121757 36058028

[B181] Zhou M. Wang X. Lin S. Liu Y. Lin J. Jiang B. (2020). Combining photothermal therapy-induced immunogenic cell death and hypoxia relief-benefited M1-phenotype macrophage polarization for cancer immunotherapy. Adv. Ther. 4 (2), 2000191. 10.1002/adtp.202000191

[B182] Zhou T. Liang X. Wang P. Hu Y. Qi Y. Jin Y. (2020). A hepatocellular carcinoma targeting nanostrategy with hypoxia-ameliorating and photothermal abilities that, combined with immunotherapy, inhibits metastasis and recurrence. ACS Nano 14 (10), 12679–12696. 10.1021/acsnano.0c01453 32909732

[B183] Zhu H. Zhang S. Ling Y. Meng G. Yang Y. Zhang W. (2015). pH-responsive hybrid quantum dots for targeting hypoxic tumor siRNA delivery. J. Control Release 220, 529–544. 10.1016/j.jconrel.2015.11.017 26590349

[B184] Zhu X. Wang M. Wang H. Ding Y. Liu Y. Fu Z. (2022). Multifunctional hollow MnO_2_ @Porphyrin@Bromelain nanoplatform for enhanced photodynamic therapy. Small 18 (52), e2204951. 10.1002/smll.202204951 36333122

[B185] Zou M.-Z. Liu W. L. Chen H. S. Bai X. F. Gao F. Ye J. J. (2021). Advances in nanomaterials for treatment of hypoxic tumor. Adv. Nanomater. Treat. hypoxic tumor 8 (2), nwaa160. 10.1093/nsr/nwaa160 PMC8288333 34691571

